# Automatic Learning of Hydrogen-Bond Fixes in the AMBER RNA Force Field

**DOI:** 10.1021/acs.jctc.2c00200

**Published:** 2022-06-14

**Authors:** Thorben Fröhlking, Vojtěch Mlýnský, Michal Janeček, Petra Kührová, Miroslav Krepl, Pavel Banáš, Jiří Šponer, Giovanni Bussi

**Affiliations:** † Scuola Internazionale Superiore di Studi Avanzati, via Bonomea 265, Trieste 34136, Italy; ‡ Institute of Biophysics of the Czech Academy of Sciences, Kralovopolska 135, Brno 612 65, Czech Republic; ∥ Department of Physical Chemistry, Faculty of Science, Palacky University, tr. 17 listopadu 12, Olomouc 771 46, Czech Republic; ⊥ Regional Centre of Advanced Technologies and Materials, Czech Advanced Technology and Research Institute (CATRIN), Palacky University Olomouc, Slechtitelu 27, 779 00 Olomouc, Czech Republic

## Abstract

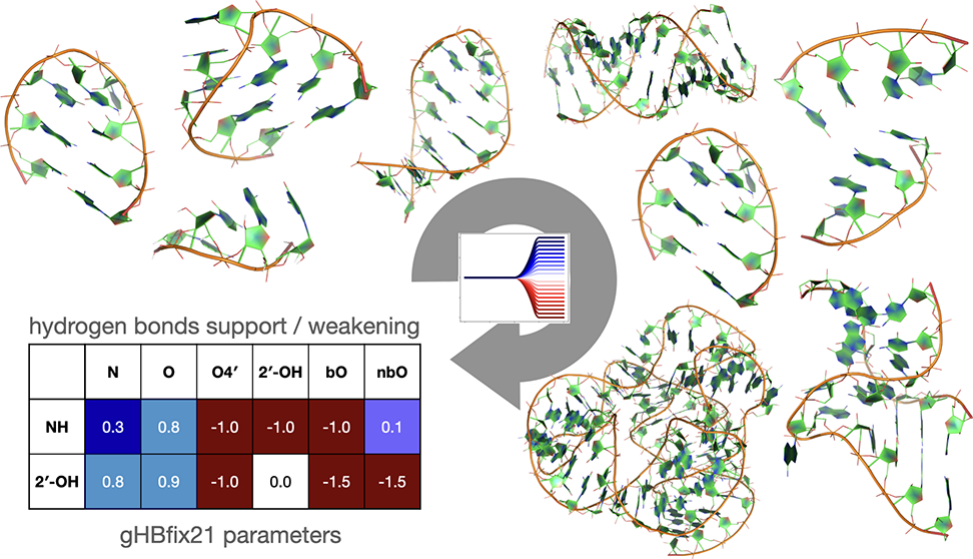

The capability of current force fields to reproduce RNA structural dynamics is limited. Several methods have been developed to take advantage of experimental data in order to enforce agreement with experiments. Here, we extend an existing framework which allows arbitrarily chosen force-field correction terms to be fitted by quantification of the discrepancy between observables back-calculated from simulation and corresponding experiments. We apply a robust regularization protocol to avoid overfitting and additionally introduce and compare a number of different regularization strategies, namely, L1, L2, Kish size, relative Kish size, and relative entropy penalties. The training set includes a GACC tetramer as well as more challenging systems, namely, gcGAGAgc and gcUUCGgc RNA tetraloops. Specific intramolecular hydrogen bonds in the AMBER RNA force field are corrected with automatically determined parameters that we call gHBfix_opt_. A validation involving a separate simulation of a system present in the training set (gcUUCGgc) and new systems not seen during training (CAAU and UUUU tetramers) displays improvements regarding the native population of the tetraloop as well as good agreement with NMR experiments for tetramers when using the new parameters. Then, we simulate folded RNAs (a kink–turn and L1 stalk rRNA) including hydrogen bond types not sufficiently present in the training set. This allows a final modification of the parameter set which is named gHBfix21 and is suggested to be applicable to a wider range of RNA systems.

## 1 Introduction

**Figure 1:**
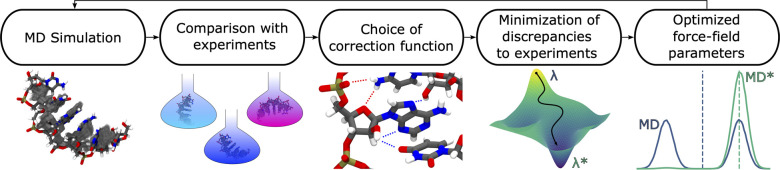
Schematic visualization of the workflow for automatic force-field refinement.^16^ After performing MD simulations on training systems for which experimental data are available, experimental quantities are back-calculated and compared with actual experimental data points. One then chooses a basis set for the correction function. gHBfix corrections^23^ are a natural choice to compensate for the possibly incorrect relative stability of hydrogen bonds in the AMBER force field. Numerical minimization is then performed so as to maximize the agreement between simulation and experiment, based on reweighting the simulated trajectories. Ideally, the resulting force field parameters enable new simulations to generate structural ensembles in better agreement with experiment also for systems not included in the training set. If necessary, a new minimization can be performed using a combination of the original and new trajectories in an iterative fashion.

**Figure 2:**
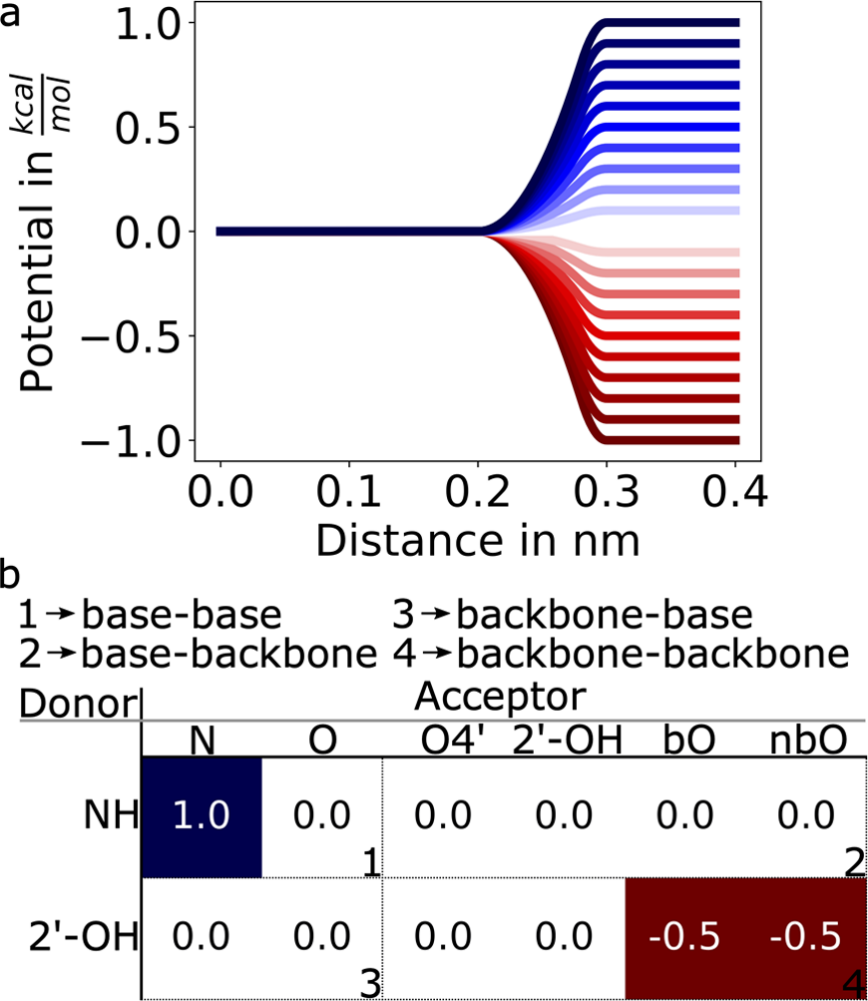
(a) Functional form for the gHBfix-correction potential,^23^ displayed as a function of the distance between a hydrogen and the corresponding acceptor. Color scale indicates corrections that could either support (blue) or disfavor (red) a hydrogen bond type. (b) In the present work, six possible acceptors and two possible donors are systematically considered, leading to a total of 12 trainable parameters. Numbers show the initial set of parameters (η parameters proposed in ref (23), also referred to as gHBfix19 parameters), expressed here as *k*_B_*T*λ in kcal/mol. Parameters are colored in blue or red according to the same scale used in panel a.

As viral pandemics are approached with RNA vaccines^1^ and RNA is becoming an increasingly relevant target in therapeutics,^2^ accurate methods for predicting and designing structure and dynamics of nucleic acids are needed to accelerate progress in these fields. Molecular dynamics (MD) simulations, in principle, allow RNA dynamics to be modeled by computing interactions using empirical force fields and directly solving the equations of motion. However, the capability of MD simulations to predict RNA dynamics is limited both by sampling issues and by force-field accuracy.^3^ Depending on the size of the system and on the complexity of the investigated conformational transitions, enhanced sampling techniques^4,5^ can help decrease the time-scale issue significantly. However, especially when long simulation time scales or enhanced sampling methods are employed, the accuracy of the underlying force fields can become a critical issue and can lead to structural ensembles that do not agree with experiment for disordered oligomers^6,7^ or for difficult structural motifs.^8,9^ A number of possible approaches can be used to take advantage of available experimental data in order to enforce agreement between experiments and simulation data^10−14^ (see also refs (15 and 16) for recent reviews and Figure 1 for a schematic). Critical and partly related issues in the application of these methods are (a) avoiding overfitting, which can be moderated by using properly tuned regularization terms,^13,16^ and (b) explicitly modeling experimental errors, which can be naturally done in Bayesian formulations.^14^ Both approaches require the degree of confidence one has in experiments and simulations to be tuned. These approaches are expected to generate transferable force fields and should not be confused with nontransferable ensemble refinements that aim at minimal ensemble corrections without requiring transferability of the resulting force-field form (see, e.g., refs (17−21)). In particular, approaches for transferable force-field refinement are dependent on the functional form of the correction terms to be fixed a priori using chemical intuition. For atomistic MD simulations, these corrections could, for instance, act on dihedral angle potentials.^11,13^ This is a natural choice, since dihedral angles are usually fitted as a last step, are expected to compensate for all of the errors accumulated in other force-field terms, and are naturally connected to the population of different rotamers.^22^ However, recent works suggested that an imbalance in the relative strength of solute–solute hydrogen bonds might be a key problem of current RNA force fields, so that fixing these terms might be more effective than acting on dihedral angles.^9,23,24^ In these works, a limited number of hydrogen-bond types were corrected using a so-called generalized hydrogen-bond fix (gHBfix, see Figure 2) with promising results. This approach allows for minimal corrections that are less likely to present side effects when compared to the more extensive reparametrization of nonbonded interactions known as the DESRES force field,^25^ as shown in ref (23). Correction factors for the gHBfix force field, leading to either supporting or disfavoring specific hydrogen-bond types, were chosen by trial and error using a protocol that might be difficult to generalize.^23^

In this paper, we expand on this idea and show that it is possible to train in an automatic fashion the correction factors associated with hydrogen-bond stabilization in the gHBfix model so as to stabilize the native structure of the difficult^9^ UUCG tetraloop structural motif. To avoid overfitting on the UUCG tetraloop, a tetraloop representative of a different class and a flexible tetramer are included in the training set. We use an approach heavily based on that reported in ref (13). As an important extension, here, we introduce and compare (a) different forms of the regularization term and (b) different protocols that can be used to perform cross-validation. Training of the 12 parameters of the gHBfix force field is done and demonstrates that this approach can lead to transferable and interpretable force-field corrections that match experimental data on a range of systems. A critical assessment of the side effects of the optimized corrections is made. Further tests on carefully chosen folded RNAs allow us to design a final set of parameters (gHBfix21) that is transferable on a wider range of RNA structural motifs. This is an upgrade of the set suggested earlier,^23^ which is also known as gHBfix19.^9^ Similarly to the preceding gHBfix19 variant, gHBfix21 should be coupled with the OL3 force field^26−29^ with modified phosphate parameters^30,31^ and OPC water model^32^ as used here.

## 2 Methods

### 2.1 Simulation Protocols

We performed simulations of several RNA systems, namely, (i) GACC, CAAU, and UUUU tetranucleotides, (ii) gcGAGAgc and gcUUCGgc 8-mer tetraloops, (iii) a ggcacUUCGgugcc 14-mer tetraloop (PDB ID 2KOC(33)), (iv) gcaccguugg (PDB ID 1QC0(34)) and uuauauauauauaa (PDB ID 1RNA(35)) RNA duplexes, and (v) kink–turn (Kt-7, PDB ID 1S72,^36^ 19 nucleotides) and L1 stalk rRNA (PDB ID 3U4M,^37^ 80 nucleotides) motifs. The starting structures of the tetranucleotides and 8-mer tetraloops (in unfolded states) were prepared using Nucleic Acid Builder of AmberTools14^38^ as one strand of an A-form duplex. The topology and coordinates of the simulated systems were prepared using the tLEaP module of the AMBER16 program package.^39^ Several trajectories for analysis were taken from our previous works (see SI Table 1 for a full list of systems and simulations). All systems were solvated using a rectangular box of OPC^32^ water molecules with a minimum distance between the box walls and the solute of 12 Å. We used the standard OL3 RNA ff^26−29^ with the vdW modification of phosphate oxygens developed in ref (30) where the affected dihedrals were adjusted as described elsewhere.^31^ The AMBER library file of this ff version can be found in the Supporting Information of ref (40). Standard MD simulations were run at ∼0.15 M KCl using the Joung–Cheatham ion parameters^41^ (K^+^: *r* = 1.705 Å, ϵ = 0.1937 kcal/mol. Cl^–^: *r* = 2.513 Å, ϵ = 0.0356 kcal/mol). Enhanced sampling simulations of the tetranucleotides and tetraloops were run at ∼0.15 and ∼1.0 M KCl salt excess, respectively. We used the hydrogen mass repartitioning scheme,^42^ allowing a 4 fs integration time step (see the Supporting Information of ref (23) for other details about the simulation protocol). Hydrogen bonds were tuned by various versions of the gHBfix potential^23^ (Table 2 in the reference and SI Table 1 of this study). Standard MD simulations were run in AMBER18,^43^ whereas both AMBER18 and GROMACS2018^44^ were used for enhanced sampling simulations. PARMED^45^ was used to convert AMBER topologies and coordinates into GROMACS inputs. Two different enhanced sampling schemes were employed, i.e., a standard replica exchange solute tempering (REST2)^46^ protocol and well-tempered Metadynamics^47−49^ (MetaD) in combination with the REST2 method (ST-MetaD).^50,51^ REST2 simulations were performed at 298 K (the reference replica) with 8 and 16 replicas for the tetranucleotides and UUCG 8-mer tetraloop, respectively. Details about the settings can be found elsewhere.^23^ The scaling factor (lambda) values ranged from 1 to 0.601700871 and from 1.0454 to 0.59984 for 8 and 16 replicas, respectively. Those values were chosen to maintain an exchange rate above 20%. The effective solute temperature ranged from 298 (8 replicas) or 285 (16 replicas) to ∼500 K. REST2 simulations were performed with the AMBER GPU MD simulation engine (pmemd.cuda).^52^ ST-MetaD simulations of both GAGA and UUCG 8-mer tetraloops were performed with 12 replicas starting from unfolded single strands and were simulated in the effective temperature range of 298–497 K for 5 μs per replica. The average acceptance rate was ∼30% for both tetraloops. The eRMSD metric^53^ was used as a biased collective variable.^8^ We used eRMSD with an augmented cutoff (set at 3.2) for biasing, which was shown to allow forces to drive the system toward and away from the native state even when nucleobases are far from each other.^8^ In a separate manuscript, we showed that using ST-MetaD with MetaD on eRMSD greatly improved the performance of pure ST for RNA tetraloops.^51^ Similar conclusions were drawn in ref (54), where parallel tempering-MetaD^55,56^ with MetaD on the number of native contacts,^57^ a variable highly correlated with eRMSD, was suggested to be significantly more efficient than pure parallel tempering for a GNRA tetraloop. ST-MetaD simulations were carried out using a GPU-capable version of GROMACS2018^44^ in combination with PLUMED 2.5^58,59^ (see section 2.7 for more details about implementation of the gHBfix function within PLUMED code and Table 1 in the Supporting Information for a full list of standard as well as enhanced sampling simulations). In theory, all replicas could be combined using a suitable reweighting procedure. However, to keep the data sets smaller, here, we decided to only analyze the reference replica of each replica-exchange simulation. Simulations for the same system performed with different force fields were combined with binless weighted-histogram analysis^60−62^ so as to maximize the statistical efficiency of the reweighting procedure.

### 2.2 Experiment-Based Force-Field Fitting

We briefly review the formalism behind experiment-based force-field fitting. Here, we used the procedure discussed in ref (13). Considering *P*_0_(*x*) as the equilibrium probability distribution of observing a conformation *x* with the original force field, the refined force field will include a correction in the form *f*(*x*,{λ}), where {λ} is a set of *N* parameters, leading to an equilibrium distribution *P*(*x*, {λ}) ∝*P*_0_(*x*)*e*^–*f*(*x*,{λ})^. Here, we assume that the correction *f*(*x*,{λ}) is a linear combination of *N* correction functions: . The modified distribution is then used to estimate the expectation value of *M* experimental observables, defined through forward models *O*_*i*_(*x*) that connect the atomic coordinates of conformation *x* with the experiment. Forward models might correspond, for instance, to Karplus equations^63^ or to indicator functions equal to 1 if *x* is a folded conformation and to 0 otherwise. Their expectation values are computed as . The cost function, to be minimized in the fitting procedure, can be written as an average of squared discrepancies between these expectation values and the corresponding experimental observations
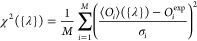
1Here, σ_*i*_ is an estimate of the experimental error associated with the *i*th data point.

In this work, the functions *f* are defined following the gHBfix potential function as formulated in ref (23). In our implementation, the parameters {λ} are unitless. However, when reporting them in figures and tables, we convert them to kcal/mol units for clarity by multiplying them by *k*_B_*T*, where *k*_B_ is the Boltzmann constant and *T* is the simulation temperature. Each of the fitted parameters thus report on how much a given hydrogen-bond type is supported (positive) or disfavored (negative).

### 2.3 Back-Calculation of Experimental Observables

The analysis was done using the same procedure used in ref (23), namely, for the tetraloops we used eRMSD^53,64^ and hydrogen bonds to identify native structures. For the tetramers, we computed the agreement with previously published NMR data.^65−67^ Exhaustive explanations can be found in the SI.

### 2.4 Fitting on Multiple Systems

The procedure above can be straightforwardly generalized to multiple systems. In practice, separate error functions are computed for each system and their linear combination is taken. Explicitly, if χ_*i*_^2^({λ}) is the error function for the *i*th system, computed using eq 1, the total cost function over *S* systems can be defined as
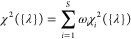
2The prefactor associated with each system in this linear combination (ω_*i*_) allows the weight of each system in the fitting procedure to be tuned. Each of the 3 systems considered in this study is assigned the same weight ω_*i*_ = 1, so that they equally contribute to the overall error. Note that these parameters have to be chosen arbitrarily and might have a significant impact on the combined χ^2^.

### 2.5 Regularization Terms

The cost function in eq 2 can be augmented with a regularization term so as to decrease the degree of overfitting
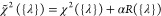
3Here, *R* is a function that takes into account how much the force field has been fitted and thus typically grows as the refined force field departs from the reference one. α is a regularization hyperparameter that can be tuned using a cross-validation procedure. Here, we compare a number of different functional forms for the regularization function *R*({λ}). The most common type of regularization is L2 regularization, where the function *R* is defined as
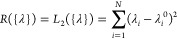
4where {λ^0^} are the parameters suggested in the original work,^23^ shown in Figure 2. This type of regularization corresponds to setting a Gaussian prior on the parameters {λ}. Indeed, the logarithm of a Gaussian function of the {λ}’s is proportional to a quadratic function of the {λ}’s. Similarly, a Laplace prior would result in a L1 regularization
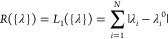
5L1 regularization leads to more sparse corrections than L2 regularization, meaning it also offers the potential to identify the most important parameters. In addition to comparing L1 and L2 regularization functions, we also tested a function that depends on the statistical significance of the generated ensemble, namely, the inverse of the Kish sample size of the reweighted trajectory^68,69^
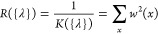
6Here, the sum runs over the whole trajectory and *w*(*x*) depends on {λ} and represents the reweighting factor for the frame with coordinates *x*, namely, , where *w*_0_(*x*) is the weight associated with the original force field, included here to take into account that simulations might have included a bias potential. We notice that a term depending on the Kish size, though different from this one, was also employed in a recent work.^14^

We then considered regularization terms that take into account the discrepancy between the prior distribution *P*_0_(*x*) and the posterior one *P*(*x*). We tested the inverse of the relative Kish size, defined as
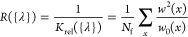
7We also considered the exponential of the negative relative entropy, defined as
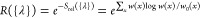
8Although these last two forms are different, they are both expected to grow as the distribution associated with the original force field and that associated with the refined force field depart from each other.

Since the last three regularization terms depend on the analyzed trajectories, they should be combined so as to take into account how each system is affected by the corrections. We decided to combine them with a LogSumExp (LSE) function
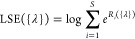
9that effectively picks the largest regularization across all systems. This makes sure that all systems have a sufficient Kish size or a sufficient similarity with the initial ensembles.

The five regularization terms discussed above were modulated by a hyperparameter α. Although in principle they could be combined, we only tested one regularization type at a time. In addition, we added boundaries for the parameter minimizations relative to the reference parameters (Figure 2). These boundaries can be interpreted as a L-infinite regularization term ( with *k*_B_*T*λ_max_ = 1 kcal/mol) used on top of one of the five regularization strategies discussed above. In practice, these boundaries avoid divergence in parameters that could otherwise become arbitrarily positive or negative.

### 2.6 Cross-Validation Strategies

The hyperparameter that tunes the regularization terms discussed in the previous section is chosen so as to maximize the performance in cross-validation. In particular, force-field corrections are fitted on a fraction of the available data set and tested on the left-out part of the data set. We performed three types of cross-validations.

(1) Cross-validation on trajectory segments: We split each trajectory in 5 segments. Here, the number of segments is chosen arbitrarily, and only the ground replica is used, which might contain spurious correlations due to replica exchanges.^70^ In principle, one might apply the splitting on continuous (demuxed) trajectories to minimize correlations and optimize the number of blocks as it is usually done in block analysis.^71^ Then, we minimize the cost function using trajectories where one of the segments was removed and finally validate the parameters by recomputing the cost function using only the left-out segments.
(2) Cross-validation on observables: We split the data set into the 7 observables (GACC: NOEs, uNOEs, and scalar couplings grouped according to the backbone angles γ (backbone1), β or ϵ (backbone2), and sugar torsional angles (sugar). GAGA and UUCG: native population) and then minimize the cost function in which the contribution of one observable is ignored and afterward validate against this left-out observable.
(3) Cross-validation on systems: We minimize the cost function only including two of the three training systems and then validate the parameters by recomputing the cost function using only the left-out system.

In all cases, the cross-validation is repeated by rotating the left-out portion of the data. The first cross-validation strategy allows one to check if the parameters would be transferable to a new trajectory simulated for the same set of systems. The other two strategies instead check the transferability to different types of observables or to different systems.

### 2.7 Implementation

To allow the gHBfix corrections to be used in generic MD codes that might not support the required functional form, we added an implementation within the PLUMED plugin^58^ that is compatible with a large number of MD packages. Specifically, a collective variable has been added that allows the user to provide two groups of atoms and then automatically compute switching functions ranging from −1 (small distance) to 0 (large distance) with a smooth interpolation in the middle. The decision to set this correction to zero for atoms at a large distance was taken to make this function compatible to other switching functions implemented in PLUMED and enable its optimization via neighbor lists. This definition is identical with that used in the original gHBfix version^40^ except for an additive constant. Multiplicative prefactors for the switching functions can be chosen based on the atom types. This collective variable can be used to analyze hydrogen-bond interactions a posteriori or to generate bias potentials to correct a simulation on-the-fly.

Here, we also updated the code “gHBfix_GenerateInput.cpp” originally published in ref (23) (https://github.com/bussilab/ghbfix-training), which is printing desired output with the newly implemented gHBfix function for PLUMED code with both required external files (typesTable.dat, scalingParameters.dat). The PLUMED input files used in this code are available on PLUMED-NEST (https://www.plumed-nest.org), the public repository of the PLUMED consortium,^72^ as plumID:21.051.

## 3 Results

**Figure 3:**
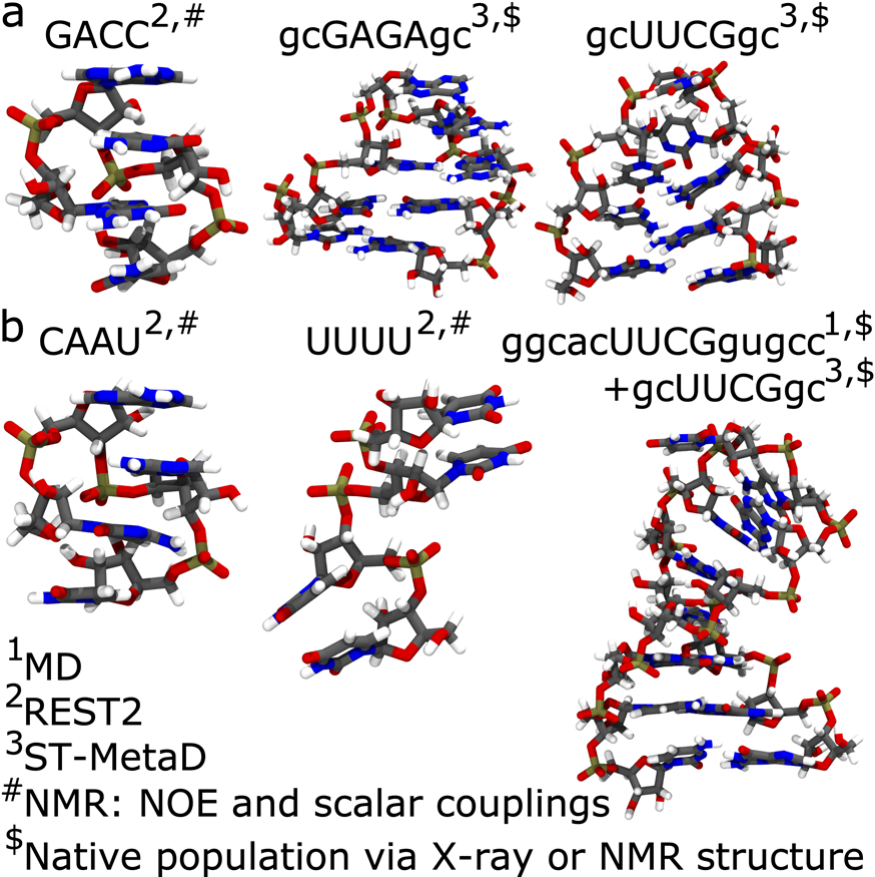
Systems used in training (a). For these three systems, we performed extensive enhanced sampling simulations. Training was done using NMR data (for GACC) or the stability of the native structure (for gcGAGAgc and gcUUCGgc). Systems used in validation (b). Quantitative validation was done using NMR data (for CAAU and UUUU) or the stability of the native structure (for gcUUCGgc), whereas qualitative validation was done running long simulations of a 14-mer containing a UUCG loop,^33^ initialized in its native structure.

Here, we train the 12 gHBfix free parameters corresponding to the 12 types of hydrogen bonds (Figure 2) by minimizing the discrepancy with respect to the experiment for three systems: two tetraloop motifs with sequences gcUUCGgc and gcGAGAgc and an oligomer with sequence GACC (Figure 3a). The two tetraloops, or similar ones, were used as a folding benchmark in a number of papers.^8,9,13,23,25,40,54,73−75^ The GACC tetramer was reported to sample intercalated structures not compatible with experiment,^7,65^ although this artifact can be significantly decreased using modified dihedral potentials^76,77^ or modified water models.^25,67,75,78^ A hyperparameter that controls overfitting is tuned by minimizing the cross-validation error. Several different forms for the regularization term are compared. Once an optimal value for the hyperparameter has been identified, a new fitting is performed including all of the training simulations, resulting in a set of optimal parameters that we refer to as gHBfix_opt_. We then test this set of parameters using additional simulations that include new systems not used during training and a new simulation of one of the systems used during training (Figure 3b). Furthermore, in order to identify the side effects of the gHBfix_opt_ parameters, we perform plain MD simulations on carefully chosen folded RNAs (kink–turn and L1 stalk rRNA). These additional simulations allow us to report a final set of parameters (gHBfix21), where one hydrogen-bond correction has been manually removed from the gHBfix_opt_ parameters, that performs well on a wider range of systems.

### 3.1 Cross-Validation Comparison

**Figure 4:**
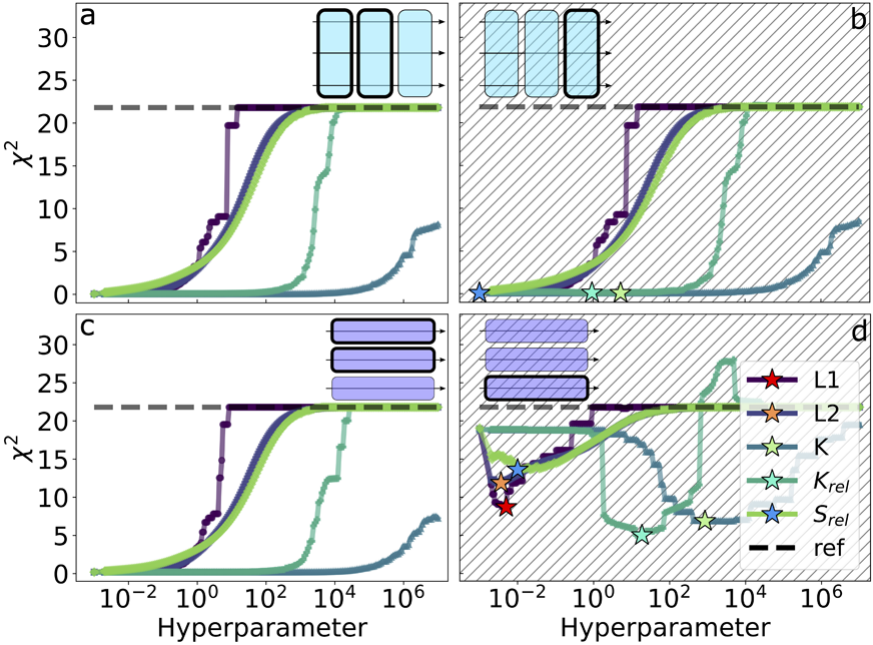
Results of the cross-validation tests on trajectory segments and on observables using all of the tested regularization methods. Error function is evaluated on the training and validation set using parameters obtained minimizing the error and a scan over a wide range of values for the regularization hyperparameter. Cyan and purple blocks show how data are split and used in cross validation. In the first case (a and b), the cross-validation is performed keeping out segments of the whole trajectories and using them as a validation set. Error is reported both for the training (a) and for the validation (b) sets. In the second case (c and d) the cross-validation is performed keeping out a fraction of the observables and using them as a validation set. Error is reported both for the training (c) and for the validation (d) set. Error function for the reference force field is reported as a dashed horizontal line. Ranges of both horizontal and vertical axes are identical.

We first perform a cross-validation test on trajectory segments. In short, we split each trajectory in 5 segments, train the 12 parameters on a subset of 4 segments, and validate against the left-out segment. We repeat the procedure five times and report the average result. We then repeat the procedure scanning the value of the regularization hyperparameter over 8 orders of magnitude and including 5 different forms for the regularization term. Figure 4a reports the average error on the training set. By construction, the error increases with the hyperparameter. For four forms of the regularization term, in the limit of large hyperparameters, the error of the original force field is recovered. When using the Kish size as a regularization term instead, this is not guaranteed. Indeed, to maximize the Kish size of the resulting ensemble, thus minimizing the regularization term, one should have uniform weights across all of the visited conformations, which is different from using the weights associated with the reference force field. The limit of low hyperparameter corresponds to fitting without any regularization. Figure 4b reports the average error on the validation set, namely, obtained using the trajectory segment that was left out during the training phase. In this case, the error systematically increases over a wide range of values of the hyperparameter. The minimum error is not appreciably different from the error obtained in the absence of regularization. This indicates that for what concerns the cross-validation on trajectory segments, there is no significant overfitting, and we should expect the obtained parameters to be transferable to a new simulation performed on the same system irrespective of regularization.

Figure 4c and 4d report a similar analysis performed by splitting all of the input data points in 7 groups corresponding to the observables, training on a subset of 6, and validating on the left-out observable. In this case, the behavior of the cross-validation error (Figure 4d) is qualitatively different. In particular, for each of the tested forms of the regularization term, we can clearly identify a specific value of the hyperparameter that minimizes the error on the validation set. When going to low values of the hyperparameter instead, we clearly see that the cross-validation error increases. This indicates that, in the absence of regularization, one would obtain parameters that would likely be nontransferable to predict new data points. The specific values of the hyperparameter that minimize the cross-validation error are shown with a star.

**Figure 5:**
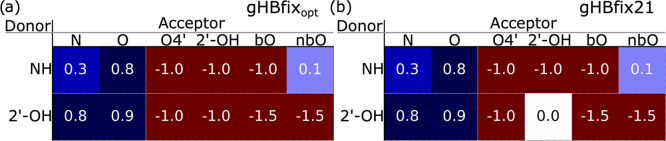
Figure representing the two sets of parameters tested in this work. (a) Parameter set referred to as gHBfix_opt_, which was obtained fitting on all of the systems in the training set using a relative Kish size regularization with a hyperparameter obtained by minimizing the error function of the cross-validation on observables. (b) Parameter set referred to as gHBfix21 in which the 2′OH–2′OH penalization is set to 0.0 in order to avoid undesirable side effects in systems with A-minor interaction and interactions with sugar–sugar H bonding. gHBfix21 parameters are those that we recommend to be used in future studies. Parameters are reported following the same convention as in Figure 2b.

We notice that given the different nature of the 5 tested regularization functions, the specific value of the hyperparameter cannot be directly compared. We can however compare the corresponding values of the cross-validation error, which suggests that the maximum transferability would be obtained using a relative Kish size regularization with a hyperparameter α = 18.68. Using this criterion to choose the type of regularization function is legitimate and is equivalent to considering the type of regularization function as an additional categorical hyperparameter that is optimized using the same cross-validation procedure. For further tests, we choose the parameters obtained with relative Kish size regularization at this optimal regularization strength. These parameters are reported in Figure 5a and referred to as gHBfix_opt_.

**Figure 6:**
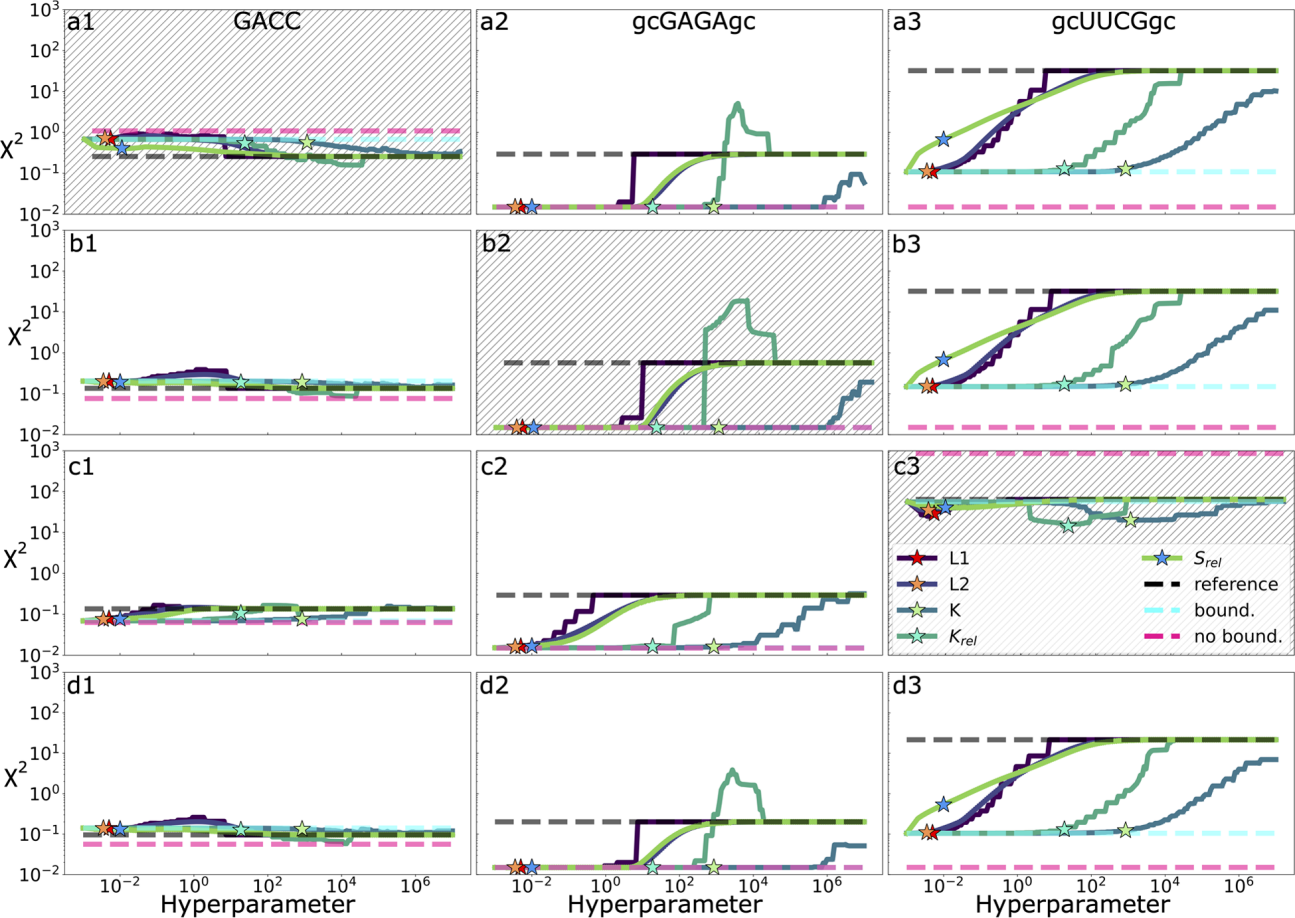
Results of the cross-validation tests on multiple systems using all of the tested regularization methods. In rows a–c the error function is evaluated on the training (white background) and validation (hatched) systems using parameters obtained minimizing the error and a scan over a wide range of values for the regularization hyperparameter. Columns 1–3 correspond to the error contribution for systems GACC, gcGAGAgc, and gcUUCGgc. Stars mark the optimal hyperparameter for the respective regularization penalty determined by cross-validation on observables (compare Figure 4). Error functions for the reference force field (gHBfix19), for an unregularized minimization without boundaries, and for an unregularized minimization with boundaries at ±1 kcal/mol relative to the original force field are reported as horizontal dashed lines. Parameters associated with each minimization obtained both with and without regularization can be found in SI Tables 3–6. Row d shows the results for the training error using all available experimental and simulation data during the fitting.

We also performed a cross-validation on systems where training is done including two systems and a validation on the left-out system (Figure 6). This analysis allows one to clearly identify the contribution of each of the three training systems to the resulting parameters. In rows a–c, the error on each of the three analyzed systems is shown, highlighting the one that was left out during training. The gcUUCGgc system has the highest error, as expected.^9^ Interestingly, gcGAGAgc is significantly improved by the presence of gcUUCGgc in the training set. However, the opposite is not true: when gcUUCGgc is excluded from training, the associated validation error displays a minimum that is almost as large as the error in the reference force field. This suggests that the gcUUCGgc native structure is stabilized by types of contact not present in the other systems. We notice that the GACC tetramer shows a light overfitting whenever gcUUCGgc is included in the training set. However, the magnitude of this overfitting is moderate, and the final χ^2^ error remains below 0.93.

It is also interesting to compare the sets of parameters obtained when regularization is present or absent and when one of the systems is left out or all three systems are used (see SI Tables 3–6). When the GACC tetramer is included in the training phase and no regularization is used, some of the parameters become very large and negative. For all considered systems, these parameters correspond to hydrogen bonds that are more abundant in the fraction of the ensemble that is less compatible with the experimental observables and thus should be penalized to improve the result. The particular importance of the concerted effect of these repulsive interactions on the correct representation of GACC can be seen in SI Figure 1, which shows that removing all repulsive interactions to the gHBfix19 parameter significantly increases the χ^2^ error of GACC to values > 1. Importantly, as soon as a regularization term is introduced, the obtained parameters are similar irrespective of which system has been left out from the training. The fit performed using all three systems with regularization also reports a similar result (compare SI Tables 3–6).

The parameters obtained in all of the tested minimizations are similar but not identical in the choice of which interactions should be disfavored and which should be supported. In particular, it emerges that NH–O base–base and 2′-OH N/O sugar–base hydrogen bonds should be supported whenever gcUUCGgc is included in training. The former type corresponds to Watson–Crick hydrogen bonds in the stem and a GU wobble pair in the loop, whereas the second type corresponds to the signature interaction of the UUCG motif.^9,79^ In SI Figure 2 one can see that all of the attractive interactions correspond to contacts that are present in the UUCG native loop. Attractive interactions 2′OH–N/O are exclusively present in the loop region and thus are particularly helpful in correctly stabilizing the challenging UUCG motif. In general, all of the hydrogen bonds formed by acceptors located in the sugar or phosphate moieties should be disfavored, with the notable exception of bonds between nonbridging oxygens and NH groups. This might be a consequence of the limited set of systems analyzed in this work, where these moieties are not involved in forming important interactions, as discussed in section 3.3.

### 3.2 Tests Using New Simulations and Additional Tetramers

The gHBfix_opt_ parameters obtained in the previous section were thus tested on new simulations. The first test simulations were performed on three systems (Table 1). In particular, we tested two other tetramers (CAAU and UUUU) for which NMR data are available. CAAU is one of the most challenging tetramers^23−25,65,67,75,77,80,81^ and shows a large χ^2^ > 5 with gHBfix19.^24^ The test simulation with gHBfix_opt_ reduces the χ^2^ value to 3.6, which is an improvement. The UUUU system is relevant since it is known to be highly dynamic in NMR experiments^65^ and was suggested to be too helical with DESRES parameters.^25^ The UUUU simulation with optimized parameters reveals a χ^2^ error of 1.48, which is also in better agreement with the experiments than the simulation performed with the gHBfix19 potential^24^ (χ^2^ of 1.63, see Table 1). These results are remarkable in that these two systems were not included in the training set. In addition, we performed new simulations to validate our improvement on the UUCG tetraloop. Specifically, a new ST-MetaD folding simulation of the gcUUCGgc system using gHBfix_opt_ corrections but otherwise identical settings as those used in the training phase resulted in a native state population of 21 ± 2%. This number is comparable with the native state population of 27 ± 4% estimated when reweighting the training simulation. Whereas this system was used during training, it is interesting that the parameters were transferable to a direct (non reweighted) simulation, consistent with what we observed in the cross-validation test on trajectory segments (see Figure 4b). In addition, we performed a qualitative test on the stability of the UUCG tetraloop by performing 10 independent 20 μs plain MD simulations of a 14-mer initialized in an NMR structure.^33^ From SI Figure 3 it can be seen that in 9 out of 10 simulations the native tetraloop structure with all of the signature interactions^9^ is stable. We prolonged the single simulation where the native state was partly lost after ∼19 μs, and we observed successful recovery of all contacts at ∼20.4 μs. The native state was then maintained until the end of this 30 μs long simulation. This is in contrast with the instability observed with other variants of the AMBER force field (see, e.g., refs (9 and 82)) and with the local conformational dynamics observed with the DESRES parameters^25^ (see refs (83 and 84)).

**Table 1:** χ^2^ Errors and Native Populations for the Training and Testing Simulations^a^

system (observable)	gHBfix19	gHBfix_*opt*_
training simulation		
GACC (χ^2^-NMR)	0.24^b^^,^^c^	0.33^e^
gcGAGAgc (native population)	24 ± 1%^d^	66 ± 3%^e^
gcUUCGgc (native population)	0.02 ± 0.002%^d^	27 ± 4%^e^
validation simulation		
gcUUCGgc (native population)	0.003 ± 0.004%^e^	21 ± 2%^d^^,^^f^
CAAU (χ^2^-NMR)	5.00^e^ (5.37^g^)	3.64^c^^,^^f^
UUUU (χ^2^-NMR)	1.73^e^ (1.63^g^)	1.48^c^^,^^f^

a For the training simulations, we report both the direct results of the simulations (gHBfix19 column) and the results predicted by reweighting those simulations to the gHBfix_opt_ parameters displayed in Figure 5a (gHBfix_*opt*_ column). Notice that for GACC the results are obtained combining simulations performed with multiple parameter sets (see text for details). For the validation simulations, we report both the direct results of the simulations (gHBfix_*opt*_ column) and the results predicted by reweighting those simulations to the gHBfix parameters with reweighting (gHBfix19 column; values in parentheses are also reporting direct results for the comparison).

b Results are obtained combining simulations performed with multiple parameter sets (see Methods for details).

c REST2 simulation.

d ST-metadynamics simulation.

e Reweighted results.

f Simulations with gHBfix_*opt*_ parameters.

g Simulations with gHBfix19 parameters.^24^

### 3.3 Tests Using Plain MD Simulations of Folded RNAs

In addition to these systems, we also choose two additional folded RNAs for which 2′OH–2′OH hydrogen bonds, which were penalized in our fitted parameters, were present in the native structure, namely, kink–turn and L1 stalk rRNA segments. The results are reported in SI Figures 4 and 5 and strongly suggest that the disfavoring of the 2′OH–2′OH contact in gHBfix_opt_ produces undesirable side effects. We thus tested a new variant where the 2′OH–2′OH term was manually set to 0.0 kcal/mol (i.e., removed). We refer to this set of parameters as gHBfix21 (see Figure 5b). This correction is critical in simulations of any systems with A-minor, phosphate-in-pocket and similar interactions with sugar–sugar H bonding. Importantly, setting to zero the 2′OH–2′OH correction does not visibly compromise the results for the systems that we used in training or validation (see SI Figure 6). Our results on the kink–turn and L1 stalk rRNA segments confirm that after removing this 2′OH–2′OH penalty no side effects are observed on their native structures. Remarkably, the test simulations also revealed that the stabilization of the 2′OH–N H bonds suggested by the fitting done on our training set further stabilizes the native kink–turn structure with respect to the uncorrected OL3 force field. Namely, the 2′OH–N term eliminates dynamical bifurcation of the most important kink–turn signature interaction between the 2′OH group of the first bulge nucleotide and N1 of the first adenine from the noncanonical stem.^85^

### 3.4 Overhead Associated to gHBfix Corrections

Simulations including gHBfix corrections suffer from a performance penalty associated with the calculation of the additional switching functions. The impact depends on the precise implementation used and, importantly, on the size of the system. Indeed, if one does not take advantage of neighbor lists, the number of pairs of atoms participating in these corrections scale with the square of the number of nucleotides. In addition, it is important to note that the relative overhead depends on other factors possibly slowing down the simulation, such as the use of metadynamics. In Table 2 we report the performance for two typical systems included in our validation set, namely, CAAU, simulated with AMBER18, and gcUUCGgc, simulated with GROMACS 2018.8 + PLUMED 2.5. For the GROMACS simulations, the reported performances were obtained using a specific implementation of gHBfix for PLUMED that is described in section 2.7 and is virtually identical with that included in PLUMED 2.8. Performances are reported for both gHBfix19 and for gHBfix_opt_ as well as for a reference calculation where no gHBfix was applied but an identical enhanced sampling protocol was used.

**Table 2:** Performance of MD Simulations Including gHBfix Terms^a^

system	software	no gHBfix	gHBfix19	gHBfix_*opt*_
CAAU (REST2)	AMBER18	712 ns/day	682 ns/day	682 ns/day
gcUUCGgc (ST-MetaD)	GROMACS + PLUMED	358 ns/day	355 ns/day	325 ns/day

a AMBER18 simulations were performed using a single core of an Intel(R) Xeon(R) E5-2620 2.10 GHz processor and a NVidia GeForce RTX 2080 Ti card per replica. GROMACS + PLUMED simulations were performed using 8 cores of an Intel(R) Xeon(R) Gold 6130 2.10 GHz processor and a NVidia GeForce GTX1080ti card per replica.

## 4 Discussion

In this work, we apply a force-field fitting strategy that was introduced in a previous work^13^ to the tuning of gHBfix hydrogen-bond interaction terms that were introduced in ref (23), obtaining parameters that we call gHBfix_opt_ here. Specifically, experimental data for two tetraloops and one tetramer are used to fit corrections that are then tested on newer simulations of one of the two tetraloops and on two tetramers not seen during fitting. The obtained parameters result in a significant stabilization of the difficult UUCG tetraloop. Since none of the RNA structures considered in the training set contain 2′OH–2′OH H bonds, we performed additional plain MD simulations of folded RNAs (kink–turn and L1 stalk rRNA) where these bonds are essential for stabilization. These additional simulations allow one to design an improved set of parameters (gHBfix21) that we suggest are applicable on a wider range of RNA systems. Using gHBfix21 instead of gHBfix_opt_ does not compromise the performance for any of the training and validation systems, while it eliminates all side effects on the native structures of kink–turn and L1 stalk rRNA. Scripts that can be used to repeat the fits and reproduce the figures of this article can be found at https://github.com/bussilab/ghbfix-training. Importantly, an implementation of the gHBfix correction for PLUMED is included in this work and has been added to PLUMED 2.8. Its impact on performance is limited, at least for short oligomers.

When compared with ref (13), we report a number of methodological improvements. First, we test five different regularization strategies. Two of them (L1 and L2) are standard in the machine learning community and can be directly interpreted as prior distributions on the parameters aimed at keeping them small (L2) or sparse (L1). We also test two additional strategies that are aimed at keeping the resulting reweighted ensemble as close as possible to the original one (relative entropy and relative Kish size). Interestingly, there is an analogy between using the relative entropy as a regularization term and the Bayesian experimental restraints introduced in ref (19). Indeed, in both cases, among the multiple possible ensembles that are equally in agreement with experiment, the method will pick the one that is as close as possible to the original ensemble. At variance with ref (19), however, the approach introduced here is aimed at deriving transferable corrections. Finally, we test the possibility to regularize using the inverse of the Kish size, which allows one to keep the resulting reweighted ensemble as statistically rich as possible. A similar idea was proposed in ref (14), though using a different functional form. We notice that in some cases the initial trajectories are generated using algorithms that provide conformations associated with a weight. This happens, for instance, when using enhanced sampling methods where a bias is applied or when combining trajectories obtained with different force fields using binless weighted histograms.^60−62^ In these cases, using a Kish size regularization makes the ensembles as uniform as possible, and thus, the result might depend significantly on which ensembles were sampled originally and which enhanced sampling strategy was used. In the last three discussed strategies (relative entropy, relative Kish size, and Kish size), the penalty introduced by the regularization term does not depend only on the parameters but also on the data and thus can be interpreted as a form of representational regularization.^86,87^

The interpretation of the prefactors associated with the gHBfix corrections^23^ is straightforward, as they directly report on how much each hydrogen-bond type is to be supported (positive coefficient) or penalized (negative coefficient). When one of the trained coefficients diverges, the frames where one interaction of that type is either present (for a negative coefficient) or absent (for a positive coefficient) are effectively removed from the ensemble. The result is thus mild depending on the exact value of the coefficients. This means that for selected training sets, one or more of the parameters might diverge with some of the regularization strategies mentioned above. This might lead to forces of infinite magnitude if these corrections were applied to a new simulation. To avoid this type of issue, we added a L-infinite-like regularization term that forces all of the parameters to be within preassigned boundaries, namely, we favor or disfavor any of the corrected pairs by at most 1 kcal/mol. The possibility to automatically repeat the training using different subsets of systems allows one to judge the contribution of each system in the overall fitting. Similarly, it is easy to repeat the training by manually removing some of the corrections, so as to identify the role of each term.

Another concept that is introduced here is that of performing a cross-validation over trajectory segments. This allows one to assess how much the parameters would be generalizable to a new trajectory for the same systems. This is a useful criterion to decrease the impact of errors due to finite sampling. In our data set, even in the absence of regularization, no significant overfitting on the trajectory segments emerges, indicating that our trajectories are long enough to be used in this training procedure. However, for more complex systems or for shorter trajectories, this might not be true.

The optimized parameters, which we refer to as gHBfix_opt_ parameters, perform well both in a new simulation of the difficult UUCG tetraloop and in the simulation of two tetramers not seen during training, confirming that the parameters are transferable. For the UUCG tetraloop, we remark that the native state population reported here is higher than that reported with DESRES parameters.^25^ Importantly, the G_L4_ bulge-out structure, which has been described both for the DESRES parameters (see refs (64, 83, 84)) and for previous variants of the AMBER force field,^7,13,74^ is not compatible with experimental solution data^33,84,88^ and is not populated in our plain MD simulations of the UUCG 14-mer. The capability of the flexible functional form of the gHBfix correction to directly stabilize the signature interactions present in the native structures with no or minimal side effects, coupled with the explicit inclusion of a UUCG tetraloop in our training set, allows for the required corrections to be automatically detected. We speculate that this result can be only achieved with such a flexible functional form. A folding simulation of the UUCG 14-mer system using the proposed parameters are left as a subject for a future work.

It is additionally important to notice that some interaction types were not present in the native structures of the systems used in our training set. These interactions were thus maximally penalized by the training procedure. Particularly relevant is the case of interactions between a pair of 2′OH groups. Sugar–sugar H bonding is an important component of A-minor and all other types of ribose zipper interactions.^3,89−92^ Sugar–sugar interactions are omnipresent in folded RNAs, and the A-minor interaction is actually the most abundant RNA tertiary interaction used by evolution.^3,93^ These interactions are indeed crucial for maintaining, for example, the native fold for a kink–turn motif and for the L1 stalk rRNA, which in turn includes two kink–turn motifs. To simulate systems where these interactions play an important role, the optimized parameters should be manually modified. In theory, one could directly include kink–turns in the training set. However, trajectories where the native structure is folded and unfolded at equilibrium would be required to estimate the effect of a correction on the stability of the native structure using a reweighting procedure. Whereas this might be possible at least for the kink–turn studied here, it would be extremely expensive and is left as a subject for a future work. Here, we decided to manually remove a single parameter and to validate it on the kink–turn motif using standard MD simulations. The resulting gHBfix21 parameter set is proposed to be applicable on a wider range of systems. This special case handling for the 2′OH–2′OH contact puts emphasis on a general problem of any fitting procedure: if a certain observation is not among those on which the method is trained, one will have to rely on extrapolation. The 2′OH–2′OH type of interaction is not formed in a significant population for any of the training systems native ensembles. Therefore, our algorithm has no way to identify undesirable effects. Only by adding extra sample systems where these interactions are crucial could we identify the problem.

We notice that with the present gHBfix_opt_ version the kink–turn and L1 stalk rRNA were significantly destabilized, however not to the same extent as with the DESRES reparametrization of the RNA force field,^25^ as shown in ref (23). This might be related to the fact that the DESRES parameters were optimized to correctly fold helices and structural motifs similar to the tetraloops used in our training set. This observation corroborates the fact that the training set should be as heterogeneous as possible to avoid overfitting.^16^ However, the flexibility of the method allows parameters to be adjusted so as to manually remove some of the terms and, if necessary, train again the remaining ones. With the adjusted gHBfix21 parameter set all side effects on the kink–turn and L1 stalk rRNA were eliminated.

An important advantage of the gHBfix functional form is its modularity, namely, the fact that it is possible to act on specific hydrogen-bond types while minimizing the indirect effects on others. In fact, it is separated from all of the other force-field terms. In order to illustrate the flexibility of the gHBfix_opt_ parameters based on some detailed system knowledge, in the SI we provide fitted force fields which are omitting specific interactions or reduce the upper and lower bounds of the parameters during fitting. In addition, the SI provides a fitting script which allows users to specify interactions to remain unchanged or within a certain magnitude and find a new force field matching these requirements (SI 8.5). In case one is concerned about too large changes of the relative stability of AU and GC pairs with the gHBfix_opt_ parameters, in SI Figure 6 we offer a gHBfix_opt_ version with a reduced magnitude of the NH–O interaction and also show its expected effects on the training set. In other words, the users could modify the gHBfix in specific projects in a system-specific manner in case the proposed gHBfix21 parameters are found to generate ensembles incompatible with some experimental information not used here. We recall that the present gHBfix21 version was derived to be applied on top of the basic AMBER OL3 RNA force field^27−29^ with phosphate oxygen corrections^30,31^ and combined with the OPC water model.^32^

Future studies should investigate whether nonlinear functions can additionally improve force fields by allowing more functional flexibility, e.g., in the form of artificial neural networks, when one attempts to find correction potentials for more extensive databases of RNA dynamics.

## Supporting Information Available

Discussion of how different experimental observables contribute to χ^2^, derivatives of the cost function, all simulations analyzed in this paper, sensitivity analysis on gHBfix_*opt*_, favored interaction types of gHBfix_*opt*_, standard simulations of the 14-mer ggcacUUCGgugcc system carried out with gHBfix19 and gHBfix_opt_, MD simulations of kink–turns Kt-7 and L1 stalk rRNA, helical properties in simulations on RNA duplexes corrected with gHBfix_*opt*_, custom constraints on corrections in order to take into account possible side effects, provided scripts (https://github.com/bussilab/ghbfix-training) allowing one to obtain all published results of this study, and parameter sets associated with the cross-validation on systems (PDF)
