# Supplementary material for: Automatic Learning of Hydrogen-Bond Fixes in the AMBER RNA Force Field
Source: J Chem Theory Comput. 2022 Jun 14;18(7):4490–502. doi: 10.1021/acs.jctc.2c00200 (PMC9281393; doi:10.1021/acs.jctc.2c00200)
Supplement: Supplementary file 1 — ct2c00200_si_001.pdf [file ct2c00200_si_001.pdf]

Supplementary information - Automatic learning of hydrogen-bond fixes in the AMBER RNA force field

Thorben Fröhlking, Vojtěch Mlýnský, Michal Janeček, Petra Kührová, Miroslav Krepl, Pavel Banáš, Jiří Šponer, Giovanni Bussi*

E-mail: bussi@sissa.it

1 Back-calculation of experimental observables

For the two tetraloops, we identified the frames corresponding to native structures using the same procedure used in Ref. 1, namely considering frames with eRMSD[2] from native smaller than 0.7[3] and all native hydrogen bonds (Watson-Crick hydrogen bonds in the stem and signature hydrogen bonds in the loop, see Figure 1 in Ref. 1). Calling p_i the population of the native state for system i, the corresponding contribution to the cost function is computed as:

$$\chi_i^2(\{\lambda\}) = \left(\log\min\left(\frac{p_i(\{\lambda\})}{0.5}, 1\right)\right)^2 . \tag{1}$$

In this manner, a penalty is added whenever the native population is lower than 50%.

For the tetramers we computed the agreement with previously published NMR data[4–6] using ^{3}J-scalar couplings, NOE and uNOE data and their corresponding forwards models. The ^{3}J-scalar couplings are obtained from relating the dihedral angles β,γ and ν_1,ν_2,ν_3 to their coupling strength (Hz) applying the Karplus relationships.[7–9] The error is computed via the coupling J, its corresponding experimental value $J_{exp,i}$ and the associated experimental uncertainty $J_{err,i}$:

$$\chi_i^2(\{\lambda\}) = \left(\frac{J_i(\{\lambda\}) - J_{exp,i}}{J_{err,i}}\right)^2 \tag{2}$$

The NOE contribution to the error considers the inter-proton distances d obtained from the NOE signal ($\frac{1}{d^6}$) converted to distance units $\bar{d} = \langle \frac{1}{d^6} \rangle^{-1/6}$. Experimental NOE distances $d_{exp,i}$, back-calculated simulation averages $\bar{d_i}$, and experimental uncertainty $d_{err,i}$ enter in the error contribution:

$$\chi_i^2(\{\lambda\}) = \left(\frac{\bar{d_i}(\{\lambda\}) - d_{exp,i}}{d_{err,i}} \right)^2 \tag{3}$$

uNOEs contribute to the error similar to NOEs, however only when their inter-proton distances are below the one experimentally expected.

$$\chi_i^2(\{\lambda\}) = \min \left(\frac{\bar{d_i}(\{\lambda\}) - d_{exp,i}}{d_{err,i}}, 0 \right)^2 \tag{4}$$

The total χ^2-error is normalized by the number of all individual observables i contributing to the error. Each of the 3 training systems considered in this study contributes equally to the error (see 2.4 in the main text).

2 Derivatives of cost function with respect to model parameters

The derivatives of the cost function with respect to the model parameters are obtained using the chain rule. We here explicitly show the derivatives of each element in the chain.

2.1 Derivatives of the observable with respect to force field parameters

The experimental observables are computed from MD trajectories of biased enhanced sampling simulations, so that each snapshot x is assigned weight $w_0(x)$. When reweighting to obtain results corresponding to a modified force field, weights should be adjusted to $w(x) \propto e^{-\sum_{j=1}^{N} \lambda_j f_j(x)}$ where N is the number of correction functions $f_j(x)$. Observables are thus computed as

$$\langle O_i \rangle = \sum_x w(x) O_i(x) \tag{5}$$

where the weights $w(x)$ should be properly normalized. It is also convenient to define the weighted averages of the basis functions used to represent the corrections:

$$\langle f_i \rangle = \sum_x w(x) f_i(x) \tag{6}$$

The derivatives of the observable with respect to the model parameters can be obtained as

$$\frac{\partial \langle O_i \rangle}{\partial \lambda_j} = -\left(\sum_x w(x) O_i(x) f_j(x) - \langle O_i \rangle \langle f_j \rangle \right) \tag{7}$$

2.2 Derivatives of the cost function with respect to the observables

In the case of tetraloops the derivative of the cost function can be written as:

$$\frac{\partial \chi_i^2}{\partial p_i} = \left(\frac{2}{p_i} \log \frac{p_i}{0.5} \right) \Theta(0.5 - p_i) \tag{8}$$

where $\Theta(x)$ is 1 if $x > 0$, 0 otherwise.

For NMR observables the cost function is a quadratic function for which the derivative is:

$$\frac{\partial \chi_i^2}{\partial O_i} = 2 \left(\frac{O_i - O_{exp,i}}{O_{err,i}} \right) \tag{9}$$

with the exception of uNOEs, for which the derivative is

$$\frac{\partial \chi_i^2}{\partial \bar{d}_i} = 2\left(\frac{\bar{d}_i - d_{exp,i}}{d_{err,i}}\right)\Theta(\bar{d}_i - d_{exp,i}) \tag{10}$$

2.3 Derivatives of the regularization terms with respect to the force field parameters

We investigate the use of L1-, L2-, Kish Size-, Relative Kish Size- and Relative Entropy-penalties. For L2 the derivative associated to it is:

$$\frac{\partial R(\{\lambda\})}{\partial \lambda_j} = \frac{\partial L_2(\{\lambda\})}{\partial \lambda_j} = 2(\lambda_j - \lambda_j^0) \tag{11}$$

For L1:

$$\frac{\partial R(\{\lambda\})}{\partial \lambda_j} = \frac{\partial L_1(\{\lambda\})}{\partial \lambda_j} = \frac{\lambda_j - \lambda_j^0}{\sqrt{(\lambda_j - \lambda_j^0)^2}} \tag{12}$$

The derivatives for the following penalties are provided for a single system where the weights of the system are $w(x) \propto e^{-\sum_{j=1}^{N} \lambda_j f_j(x)}$. The inverse Kish Size derivative follows as:

$$\frac{\partial R(\{\lambda\})}{\partial \lambda_j} = \frac{\partial K(\{\lambda\})}{\partial \lambda_j} = -2\sum_x w^2(x)(f_j(x) - \langle f_j \rangle) \tag{13}$$

For Relative Kish Size the derivative changes to:

$$\frac{\partial R(\{\lambda\})}{\partial \lambda_j} = \frac{\partial K_{rel}(\{\lambda\})}{\partial \lambda_j} = -2\sum_x \frac{w^2(x)}{w_0(x)}(f_j(x) - \langle f_j \rangle) \tag{14}$$

For Relative Entropy where $-S_{rel} = \sum_x w(x) \log \frac{w(x)}{w_0(x)}$ the derivative can be written as:

$$\frac{\partial R(\{\lambda\})}{\partial \lambda_j} = \frac{\partial e^{-S_{rel}(\{\lambda\})}}{\partial \lambda_j} = -e^{-S_{rel}(\{\lambda\})}\sum_x w(x) \log \frac{w(x)}{w_0(x)}(f_j(x) - \langle f_j \rangle) \tag{15}$$

For the regularization penalties Kish Size, Relative Kish Size and Relative Entropy, a LogSumExp (LSE) is employed such that the system with the largest penalty contributes to the χ^2 and therefore it is useful to consider its derivative with respect to the penalty of each system:

$$\frac{\partial LSE(\{R\})}{\partial R_i} = \frac{e^{R_i}}{\sum_{j=1}^{S} e^{R_j}} \tag{16}$$

3 Additional simulation details

An overview of all the simulations analyzed in this paper is reported in Table S1. Notice that some of the simulations were taken from previous publications. For reproducibility, all input files are provided on Zenodo and on GitHub (see links in the main text). PLUMED input files, used for enhanced sampling, are also available on the PLUMED-NEST.[10]

Table S1: Overview of simulations of RNA systems considered in this study[a].

Method [b]	System (PDB ID)	gHBfix variants [c]	# of independent simulations/ # of replicas	Simulation length (μs)	Cumulative time (μs)
ST-MetaD	gcGAGAgc	gHBfix$_{0.5-0.5}$	1 / 12	5	60[A]
ST-MetaD	gcGAGAgc	gHBfix19	1 / 12	5	60[A]
ST-MetaD	gcUUCGgc	gHBfix$_{0.5-0.5}$	1 / 12	5	60[A]
ST-MetaD	gcUUCGgc	gHBfix19	1 / 12	5	60[A]
ST-MetaD	gcUUCGgc	gHBfix$_{opt}$	1 / 12	5	60[C]
REST2	GACC	gHBfix(x) [d]	14 / 8	10	1120[B]
REST2	CAAU	gHBfix19	1 / 8	10	80[B]
REST2	UUUU	gHBfix19	1 / 8	10	80[B]
REST2	CAAU	gHBfix$_{opt}$	1 / 8	10	80[C]
REST2	UUUU	gHBfix$_{opt}$	1 / 8	10	80[C]
MD	UUCG 14-mer (2KOC)	gHBfix19	10 / -	20	200[A,C]
MD	UUCG 14-mer (2KOC)	gHBfix$_{opt}$	10 / -	20 (30) [e]	210[C]
MD	RNA duplex (1QC0)	gHBfix$_{opt}$	2 / -	5	10[C]
MD	RNA duplex (1RNA)	gHBfix$_{opt}$	2 / -	5	10[C]
MD	Kt-7 (1S72) [f]	gHBfix$_{opt}$	3 / -	2	6[C]
MD	Kt-7 (1S72) [f]	gHBfix21[g]	3 / -	2	6[C]
MD	L1 stalk rRNA (3U4M) [h]	gHBfix$_{opt}$	3 / -	1	3[C]
MD	L1 stalk rRNA (3U4M) [h]	gHBfix21[g]	3 / -	1	3[C]

[a] Simulations performed with χOL3CP-RNA-ff and specific versions of gHBfix potential (see Methods)
[b] We used standard MD simulations (MD) and two different enhanced sampling protocols, namely, (i) standard replica exchange solute tempering (REST2) and (ii) combination of REST2 with well-tempered metadynamics (ST-MetaD).
[c] Different Base–base interactions, i.e., either only –NH. . . N– interactions were stabilized by 1.0 kcal/mol ("standard" version of the gHBfix potential labelled as gHBfix19)[1] or both –NH. . . N– and –NH. . . O– interactions were stabilized by 0.5 kcal/mol (labeled as gHBfix$_{0.5-0.5}$). We also weakened sugar donor-phosphate acceptor H-bonds (i.e., –OH. . . bO/nbO– interactions) by 0.5 kcal/mol in both versions.
[d] 14 different gHBfix variants as tested in the original work (see Ref. 1).
[e] Nine simulations with length 20 μs and one simulation with length 30 μs.
[f] Simulated Kt-7 structure was obtained by excising the residues 76-83 and 91-101 from the structure of the large ribosomal subunit of *Haloarcula marismortui*. The Kt-7 is known to fold autonomously into the tightly-kinked structure in solution even in absence of divalent ions and proteins.
[g] Correction to 2′OH-2′OH contact set to 0.0 kcal/mol.
[h] Highly-conserved RNA medium-sized (80 nucleotides) ribosomal RNA segment that contains several RNA motifs and diverse tertiary interactions. It adopts the folded structure even in isolation in the absence of the ribosomal context.
[A] Data taken from Ref. 11.
[B] Data taken from Ref. 1.
[C] New simulations performed for this work.

4 Analysis of individual interaction types

Donor	NH						2'-OH						Parameters	System (observable)		
Acceptor	N	O	O4'	2'-OH	bO	nbO	N	O	O4'	2'-OH	bO	nbO		gcGAGAgc (native population)	gcUUCGgc (native population)	GACC (χ^2-NMR)
	1.0	0.0	0.0	0.0	0.0	0.0	0.0	0.0	0.0	0.0	-0.5	-0.5	gHBfix19	24%	0.02%	0.24
	0.3	0.8	-1.0	-1.0	-1.0	0.1	0.8	0.9	-1.0	-1.0	-1.5	-1.5	gHBfix$_{opt}$	66%	27%	0.33
													**Individual interactions constrained to gHBfix19**			
	1.0*	0.0*	-1.0	-1.0	-1.0	0.0*	0.0*	0.0*	-1.0	-1.0	-1.5	-1.5	All attractive interactions	35%	0.25%	0.18
	1.2	0.6	0.0*	0.0*	0.0*	0.2	0.9	1.0	0.0*	0.0*	-0.5*	-0.5*	All repulsive interactions	39%	2%	1.43
	1.0*	0.7	-1.0	-1.0	-1.0	0.0	0.8	1.0	-1.0	-1.0	-1.5	-1.5	NH-N	68%	27%	0.50
	1.5	0.0*	-1.0	-1.0	-1.0	-0.5	1.0	0.8	-1.0	-1.0	-1.5	-1.5	NH-O	29%	3%	0.43
	0.3	0.8	0.0*	-1.0	-1.0	0.1	0.9	0.9	-1.0	-1.0	-1.5	-1.5	NH-O4'	58%	26%	0.39
	0.5	0.7	-1.0	0.0*	-1.0	0.1	0.8	0.9	-1.0	-1.0	-1.5	-1.5	NH-2'OH	65%	19%	0.71
	0.4	0.8	-1.0	-1.0	0.0*	0.1	0.9	0.9	-1.0	-1.0	-1.5	-1.5	NH-bO	63%	27%	0.35
	0.3	0.8	-1.0	-1.0	-1.0	0.0*	0.8	0.9	-1.0	-1.0	-1.5	-1.5	NH-nbO	64%	27%	0.32
	0.4	0.9	-1.0	-1.0	-1.0	0.1	0.0*	1.0	-1.0	-1.0	-1.5	-1.5	2'OH-N	53%	16%	0.24
	0.3	0.9	-1.0	-1.0	-1.0	0.3	1.0	0.0*	-1.0	-1.0	-1.5	-1.5	2'OH-O	67%	18%	0.43
	0.6	0.7	-1.0	-1.0	-1.0	0.2	1.0	0.9	0.0*	-1.0	-1.5	-1.5	2'OH-O4'	70%	19%	0.57
	0.5	0.7	-1.0	-1.0	-1.0	0.2	0.9	0.9	-1.0	0.0*	-1.5	-1.5	2'OH-2'OH	68%	25%	0.38
	0.6	0.7	-1.0	-1.0	-1.0	0.1	1.0	1.0	-1.0	-1.0	-0.5*	-1.5	2'OH-bO	66%	20%	0.44
	0.5	0.8	-1.0	-1.0	-1.0	0.2	0.9	0.9	-1.0	-1.0	-1.5	-0.5*	2'OH-nbO	66%	26%	0.39

Figure S1: Sensitivity analysis on the tunable parameters of the gHBfix model. Specifically, we constrained one parameter at a time to its value in the gHBfix19 model (indicated by a star), which is zero in 9 bond types, 1 kcal/mol for NH-N types, and -0.5 kcal/mol for $2'$-OH-phosphate bonds. Then we reoptimized the 11 remaining parameters. Most of the parameters magnitudes are invariant to the constraining of a single interaction to the gHBfix19 value. This is especially important for the $2'$OH-$2'$OH interaction, which is constrained to zero for gHBfix21 in order to make corrections transferable to the kink-turn and L1 stalk rRNA (compare main text). Important exceptions from this general trend are NH-N and NH-O, which are the Watson-Crick interactions stabilizing duplex regions. The NH-O interaction has larger effect on the stability of the folded state than NH-N, because it appears twice per GC base pair and omitting this term leads to a significant drop in native populations of the tetraloops. When NH-O is constrained to zero, NH-N reaches the boundary value (1 kcal/mol) to compensate the missing stabilization, but still results in a significant decrease of the tetraloops stabilities. We also report the results of optimizations where either all attractive or all repulsive corrections (*i.e.*, respectively, larger and lower than gHBfix19) are constrained. Without repulsive interactions, an important increase in χ^2-error of GACC is observed. This is in line with the finding of significantly larger repulsive interactions during the cross-validation on systems when GACC is included in the training and no regularization is used (see main text). This indicates that the concerted effect of these repulsive interactions is crucial for correctly representing the unstructured GACC ensemble. In both cases (not allowing attractive or not allowing repulsive corrections), the predicted stability of the UUCG tetraloop is significantly decreased, whereas the impact on the stability of the GAGA tetraloop is milder.

5 Attractive interactions in the challenging UUCG motif

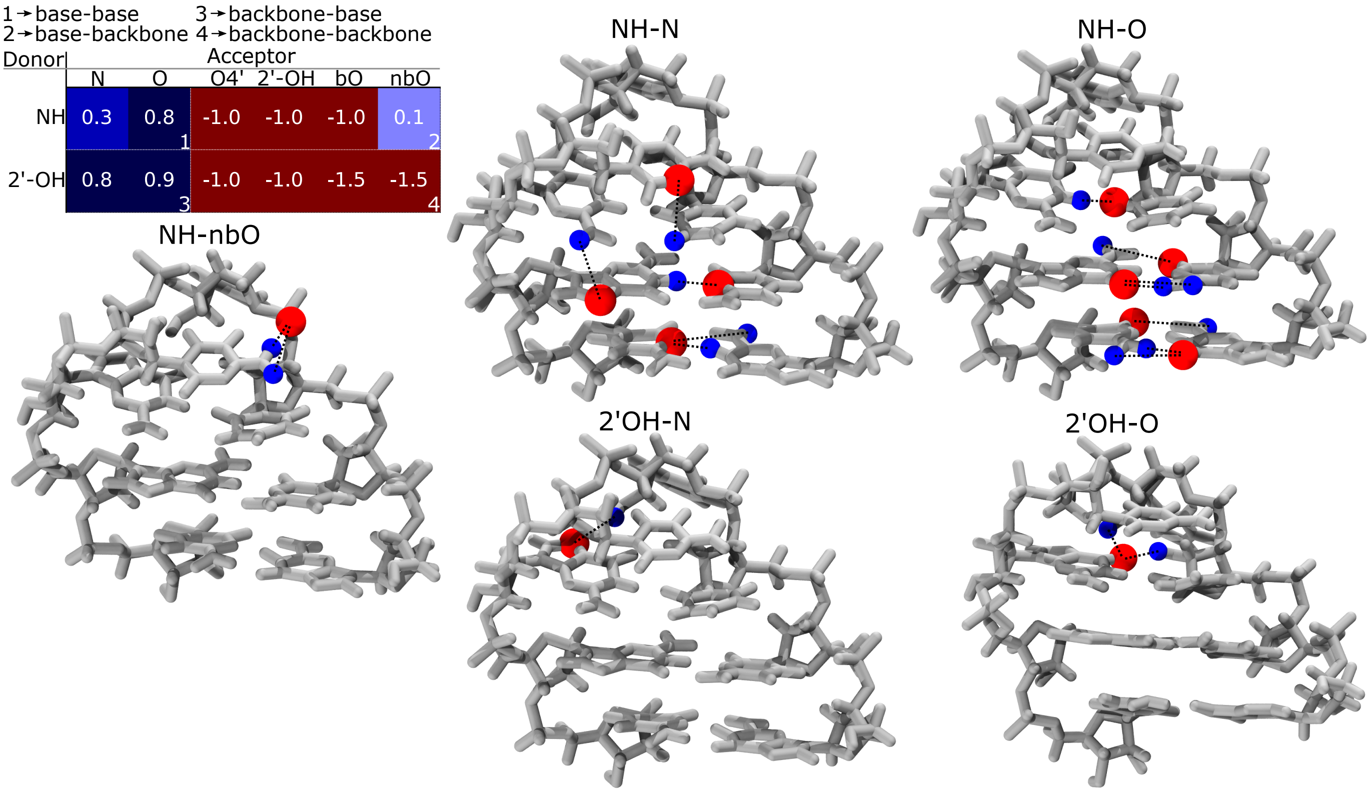

Figure S2: Parameter set gHBfix$_{opt}$, top left panel. The five interaction types that are favored with respect to the standard AMBER force field are shown in blue. In the remaining panels, atoms forming these interaction types in the native structure of the gcUUCGgc tetraloop are visualized. Hydrogen bond donors (blue) and hydrogen bond acceptors (red) of the respective interaction are shown as spheres. NH-N and NH-O bonds stabilize the stem. NH-NbO interactions are subject to a very limited stabilization (0.1 kcal/mol), whereas the most sizable attractive interactions ($2'$OH-N and $2'$OH-O) are exclusively present in the loop region.

6 Validation MD on system containing the UUCG motif

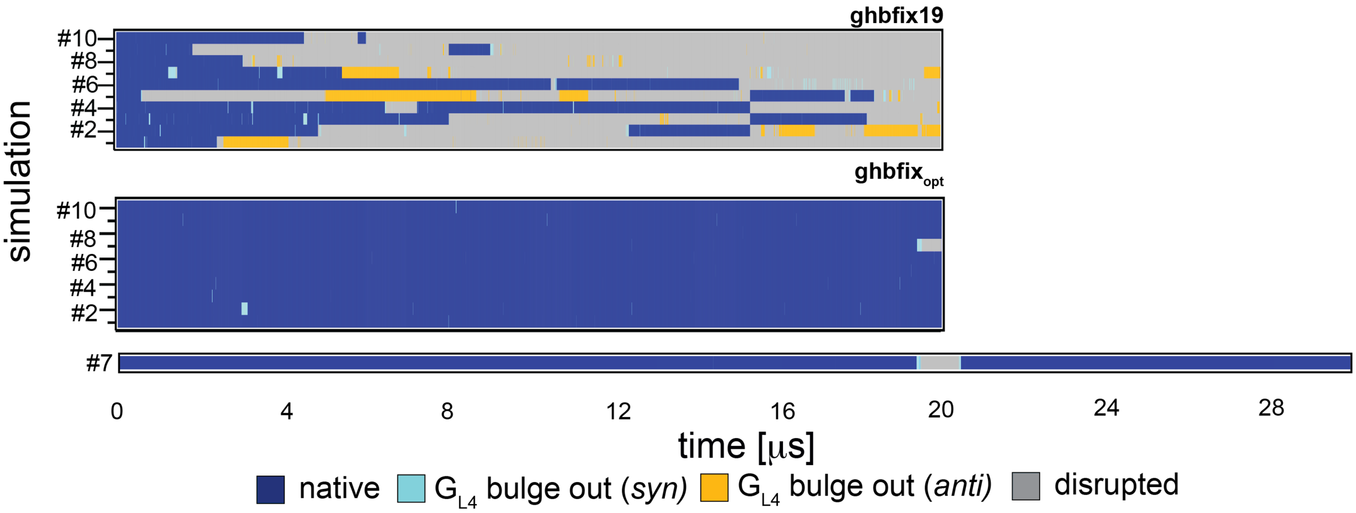

Figure S3: Summary of standard simulations of the 14-mer ggcacUUCGgugcc system carried out with gHBfix19 and gHBfix$_{opt}$ parameter sets, starting from the folded state using the high-resolution 2KOC NMR structure.[12] With both gHBfix versions, we collected 10 independent 20 μs-long standard MD simulations and monitored the structural stability of the UUCG tetraloop, differentiating four states, namely: native state with all signature interactions of the tetraloop satisfied, state with the GL4 nucleotide bulged out but remaining in the either *syn* or *anti* conformation, and completely disrupted UUCG architecture, similarly to Ref. 13. The canonical stem below the tetraloop was entirely stable in all simulations. With the gHBfix19, the native UUCG fold is progressively lost in the simulation set. In contrast, with gHBfix$_{opt}$, the folded state remained stable in 9 simulations while it has been lost at the end of simulation '#7'. We have thus prolonged this simulation up to 30 μs (lower panel), and observed that the native structure was recovered at $\approx$ 20.4 μs and stable till the end of the simulation. These results are in line with the finding of significant increase of native population in the validation gHBfix$_{opt}$ ST-MetaD simulation for the shorter gcUUCGgc system. The standard simulations reveal dramatic increase of structural stability (lifetime) of the folded UUCG tetraloop by gHBfix$_{opt}$ compared to gHBfix19.

7 MD simulations of kink-turns with gHBfix$_{opt}$ reveal instabilities related to the 2′OH-2′OH correction while significantly improving the signature interaction

We simulated an isolated Kink-turn, a recurrent RNA motif widespread in the ribosome, which facilitates a sharp bend (kink) in the canonical A-RNA helix.[14–16] Kink-turns possess a highly conserved and intricate topology forming a characteristic bulge with typically three-nucleotides. The bulge is flanked by a non-canonical (NC) stem, containing at least two consecutive trans Hoogsteen/Sugar edge A-G base pairs and a canonical stem with cis Watson-Crick/Watson-Crick G-C base pairs. An A-minor interaction between the two stems stabilizes the sharp bend. Furthermore, there is a signature 2′OH–N1 H-bond between the first nucleotide of the bulge and adenine from the A-G base pair closest to the bend. This interaction has been experimentally identified as the single most important interaction stabilizing kink-turns.[17] Since the stability of the kink-turn motif is dependent on simultaneous presence of multiple NC RNA interactions, it is an ideal system for verifying that new force-field corrections are not overfitted towards canonical A-form. The motif also exhibits relevant dynamics as it can function as a molecular elbow, facilitating changes in orientation and absorbing strains from connecting A-RNA helices.[18–20] We simulate the structure of Kink-turn 7 (Kt-7) from the ribosome of *Haloarcula marismortui*, which has a sequence close to a consensual kink-turn motif. Kt-7 also requires neither proteins nor divalent ions to adopt a tightly kinked geometry.[21,22] In the simulation of Kt-7 using gHBfix$_{opt}$, we observe a notable improvement in the behavior of signature 2′OH–N1 H-bond whose low stability was so far a persistent problem in kink-turn simulations. Namely, with standard force-fields this interaction tends to fluctuate between the native signature interaction and a non-native 2′OH–N6 H-bond.[23] To our knowledge, all experimental structures indicate presence of the 2′OH–N1 H-bond. Yet, for Kt-7, standard force fields predict a non-native population of around 50%, indicating a force field problem. The newly parametrized gHBfix$_{opt}$ potential penalizes the NH–2′OH while supporting N–2′OH, which significantly stabilizes the native signature interaction in Kt-7 as shown in Fig. S4.

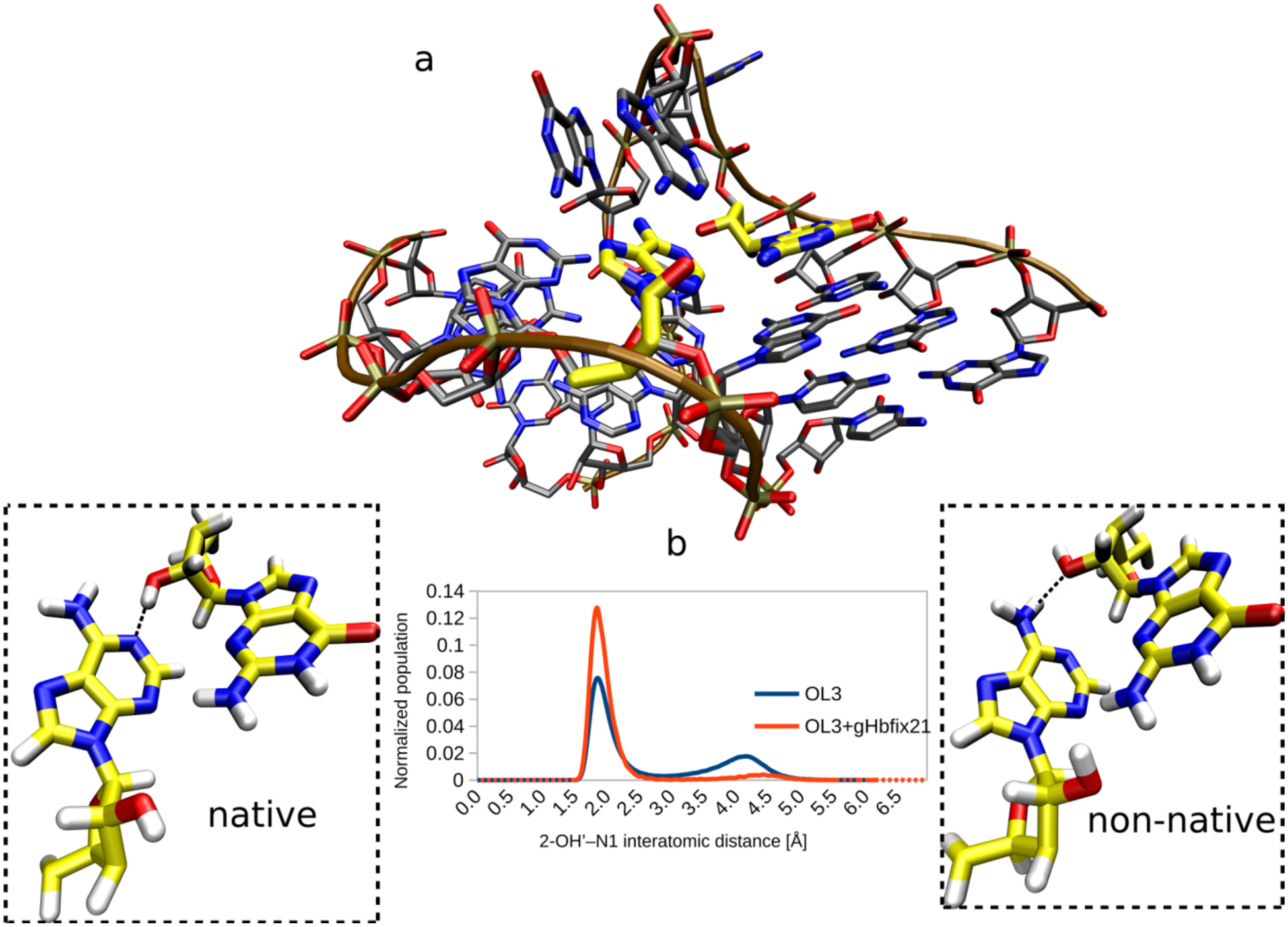

Figure S4: Simulation results using gHBfix21. a) The nucleotides in Kink-turn 7 form the signature 2′OH–N1 H-bond interaction highlighted by yellow carbons. b) Native (left) and non-native (right) states of the signature interaction observed in MD simulations. As shown in the histogram of 2′OH–H1-N1 H-bond distances (middle) gHBfix21 significantly suppresses population of the non-native H-bond variant compared to a standard force field.

While significantly improving description of the signature interaction, the kink-turn simulations also revealed instability of the A-minor interaction when applying the gHBfix$_{opt}$ corrections. This can be attributed to the -1.0 kcal/mol penalization of the 2′OH–2′OH interaction which is involved in the A-minor interaction (Fig. S5). From the start of the simulations the 2′OH–2′OH interaction is unstable and a transition from A-minor I to A-minor 0 can be observed in all simulations. This transition occurs with the standard force field as well and we do not consider it a force-field problem since both types of A-minor interactions are known in kink-turns.[24] The preference for type I or type 0 is largely dictated by the surrounding segments, such as bound proteins[25] and is altered when simulating isolated Kt-7 out of its native structural context. However, since the 2′OH–2′OH interaction occurs in A-minor 0 as well, as can be seen from Fig. S5, the simulation instabilities eventually progressed, leading to a complete loss of A-minor interaction in all three simula-

tions. In one of the simulations, this even led to a permanent loss of the sharp bend in the kink-turn (see Fig. S5). Therefore penalization of the 2′OH–2′OH interaction, although providing benefits for other systems, is problematic for the kink-turn. Consequently we tested the gHBfix21 parameters, where the penalty on the 2′OH–2′OH interaction has been omitted. In these simulations we observed A-minor I remaining stable in one simulation, as well as successful transitions into A-minor 0 in the two other simulations with no tendency for straightening of the kink-turn. The other segments and interactions remained stable as well. Importantly, the benefit of the improved signature 2′OH–N1 H-bond interaction provided by gHBfix$_{opt}$ was maintained in these simulations. The stability of the native state was at over 90% compared to 60% when using the standard force field (Fig. S4). As a result of this detailed analysis, we suggest that gHBfix$_{opt}$ can provide strong benefits in simulations of kink-turns, however penalization of the 2′OH–2′OH interaction needs to be avoided in this case. The gHBfix21 parameters, where the penalty on the 2′OH–2′OH interaction has been omitted, are more transferable to any systems with A-minor interactions and interactions with sugar-sugar H-bonding.

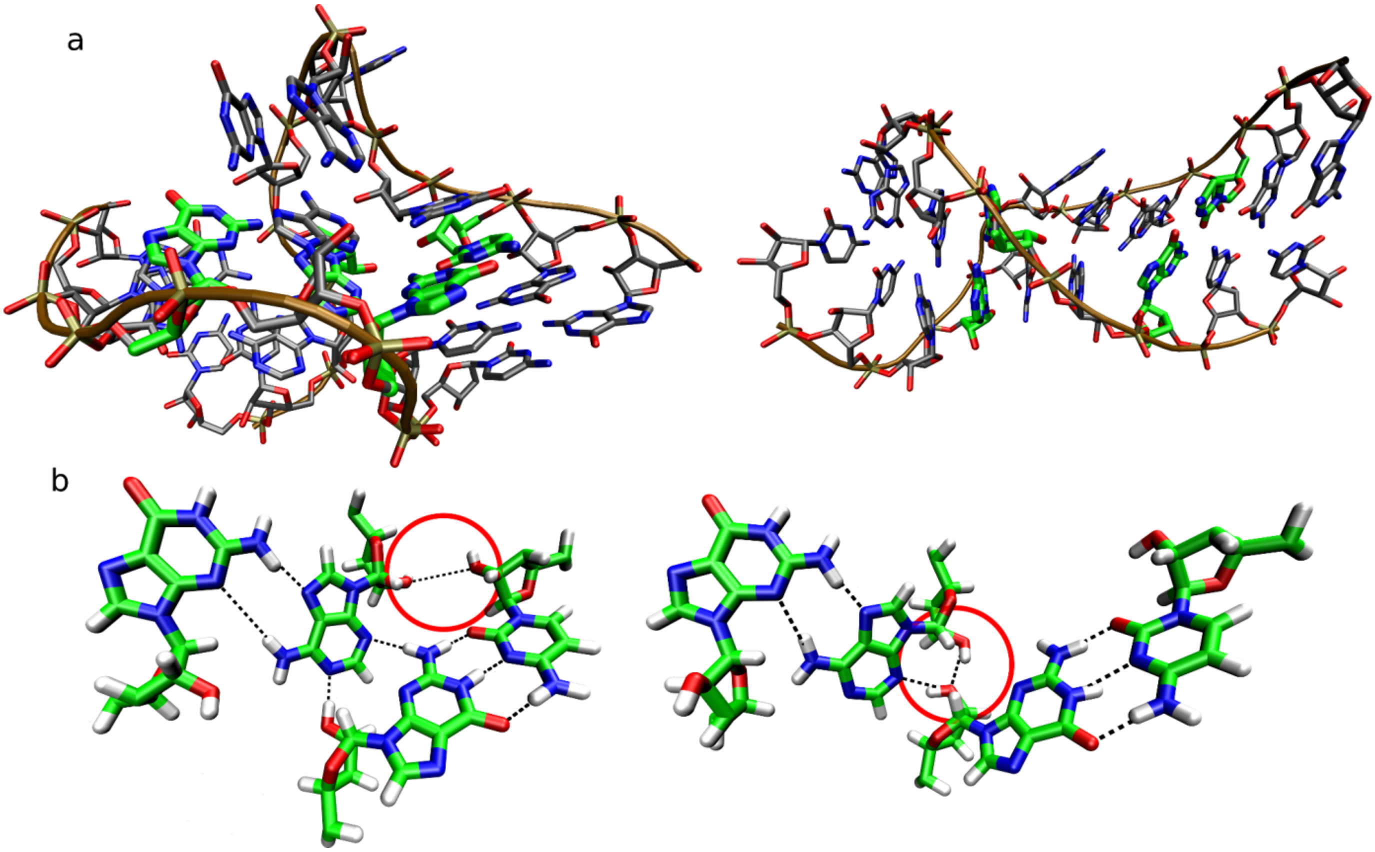

Figure S5: Structures observed in the simulations of the kink-turn. a) Nucleotides forming the A-minor interaction (left) are highlighted with green carbons. In simulations in which the 2′OH–2′OH interaction was corrected by -1.0 kcal/mol we observed instability of the A-minor interaction and sometimes even a loss of the kink-turn (right). b) The 2′OH–2′OH interaction (red circles) is prominent in both A-minor I (left) and A-minor 0 (right) conformations.

Next, we simulated structure of the L1 stalk rRNA of the large ribosomal subunit. The L1 stalk rRNA is a highly conserved and mobile element which facilitates exit of the deacylated tRNA from the ribosome during the elongation cycle.[26,27] The L1 stalk rRNA contains a multitude of NC interactions and RNA recurrent motifs, making it yet another useful model for force-field validation. Namely there are two kink-turns, a UCCG tetraloop and a structured internal loop.[28] Together these elements form a highly intricate six-layered nucleotide platform composed of NC base pairs and triplets stacked on top of each other. At the top of this highly-conserved platform is the binding site for the deacylated tRNA.[29] Previous simulations showed that the L1 stalk rRNA can be well described using standard RNA force fields.[23] Our motivation was therefore to verify that this performance is not deteriorated by applying gHBfix$_{opt}$. Similar to the Kt-7 simulations the two kink-turns Kt-77 and Kt-78 within the L1 stalk rRNA were less stable using gHBfix$_{opt}$ corrections. In case of Kt-77 instabilities were minor and reversible as this kink-turn is firmly held in place by the surrounding RNA segments. However increased fluctuations of its A-minor interaction compared to the standard force field were noticeable. In case of Kt-78, which is more dynamic, we have observed complete loss of its A-minor interaction in all three performed simulations. However unlike Kt-7, the kink-turn did not completely straighten. The NC segments, such as the UCCG tetraloop, the internal loop and the nucleotide platform were described on par with the standard force field. We noticed a modest increase of fluctuations among the native 2′OH–phosphate interactions, but this did not pose a problem on the timescale of the simulation. In conclusion, also the simulations of the L1 stalk rRNA suggest that the 2′OH–2′OH penalization should be avoided for RNA structures containing kink-turns as this motif relies on the stability of these interactions. Although the performance of gHBfix$_{opt}$ for the L1 stalk rRNA is not fully satisfactory, issues were only moderate and visible only after detailed analysis. We note that there was no global degradation of its fold, which was previously reported in simulations using the DESRES force field.[1] Importantly, the significant improvement of the 2′OH–N1 signature interaction was also observed for both kink-turns in the L1 stalk rRNA. When simulating with gHBfix21 (i.e. without penalization of the 2′OH–2′OH contact), both Kt-77 and Kt-78 were fully stable and the simulation performance for L1 stalk rRNA can be considered superior to the standard force field.

8 Impact on helical properties

Averages of helical parameters (see Table S2) in simulations on both RNA duplexes corrected with gHBfix$_{opt}$ potential are comparable to averages obtained from simulations with gHBfix19.

Table S2: Comparison of helical parameters (mean values and standard deviations represented by error bars) between experiments (X-ray structures) and MD simulations of RNA duplexes (1RNA, 1QC0) [a].

**Exp. / gHBfix version**	**Inclination [°]**	**Slide [°]**	**Propeller [°]**	**Roll [°]**	**Minor groove [Å]**	**Major groove [Å]**
X-ray values (1QC0)	15.2	-1.7	-12.5	8.1	13.1	18.1
gHBfix19 (1QC0)	14.9 ± 0.7	-1.8 ± 0.1	-12.9 ± 0.7	8.1 ± 0.4	13.38 ± 0.01	19.18 ± 0.03
gHBfix$_{opt}$ (1QC0)	13.7 ± 0.8	-1.87 ± 0.04	-12.0 ± 0.7	7.4 ± 0.5	13.40 ± 0.02	19.17 ± 0.04
	13.9 ± 0.7	-1.87 ± 0.04	-12.2 ± 0.6	7.5 ± 0.4	13.40 ± 0.02	19.17 ± 0.04
X-ray values (1RNA)	18.8	-1.3	-18.8	10.0	13.3	18.3
gHBfix19 (1RNA)	18.1 ± 0.5	-1.49 ± 0.02	-17.5 ± 0.2	10.5 ± 0.3	13.29 ± 0.02	19.22 ± 0.02
gHBfix$_{opt}$ (1RNA)	18.4 ± 0.6	-1.50 ± 0.03	-17.5 ± 0.5	10.7 ± 0.3	13.30 ± 0.01	19.21 ± 0.05
	18.4 ± 0.7	-1.50 ± 0.03	-17.4 ± 0.8	10.7 ± 0.4	13.29 ± 0.04	19.2 ± 0.1

[a] All simulations were run with χOL3CP-RNA-ff with OPC water model and 0.15 M KCl using Joung-Cheatham ion parameters. We compared behavior of simulations using the gHBfix19 potential[1] and gHBfix$_{opt}$ (two independent simulations, parameters developed in this work with methods, which can be found in the main text). Two terminal base pairs from both each duplex ends were excluded from analysis.

9 Custom constraints on corrections

In order to take into account possible side-effects of the gHBfix corrections we constrained interactions of interest or performed refitting of the training data set with additional constraints and we collected the expected effect on the training as well as the validation systems in Fig. S6. The sets of constrained parameters are manually chosen to test relevant hypothesis, and the other parameters are either left unchanged or optimized again so as to maximise the performance on the training set.

Specifically, we tested the effect of removing the penalty on 2′OH-2′OH and on NH-2′OH bonds, that might be relevant for maintaining the native structures in folded RNAs. In the three tested combinations we observe that (a) the impact on the training set is mostly observed in a decrease native population of the gcUUCGgc tetraloop, that can be moderated by reoptimizing the remaining parameters and (b) the effect of the additional optimization on the validation set is limited or detrimental. This indicates that this further training leads to overfitting on the training set. A possible interpretation is that the removed bonds provide the only possible way to improve the native populations, by destabilizing non-native gcUUCGgc structures, and other gHBfix corrections cannot compensate for this removal without resulting in overfitting. In these cases, thus, the simplest approach of manually removing one or more correction term *without reoptimization* should be considered as valid. We also notice that the removal of only 2′OH-2′OH corrections, that we here tested on the Kt-7 and L1 stalk rRNA systems, has a very limited predicted impact on the native gcUUCGgc population.

We then notice that the relative pairing energy of AU and GC pairs have been reported to be in agreement with experiments,[30,31] although using a water model different from the one employed here. Hence we test the case where NH-O bonds are constrained to avoid modifying too much the difference between different Watson-Crick pairs in the original force field. This is obtained by setting NH-O bonds, which contribute to the difference in AU vs GC canonical pairs, to a maximum of 0.5 kcal/mol. In this case, a compensating increase of the NH-N stabilization can largely compensate for this constrain. In this case, there is no detrimental effect on the gcUUCGgc test simulation (last column), and only a moderate increase of the CAAU χ^2-error.

Donor: NH						Donor: 2'-OH						Parameters	System					
Acceptor: N	O	O4'	2'-OH	bO	nbO	N	O	O4'	2'-OH	bO	nbO		gcGAGAgc	gcUUCGgc	GACC	CAAU	UUUU	gcUUCGgc
1.0	0.0	0.0	0.0	0.0	0.0	0.0	0.0	0.0	0.0	-0.5	-0.5	gHBfix19	24%	0.02%	0.24	5.00	1.73	0.003%
0.3	0.8	-1.0	-1.0	-1.0	0.1	0.8	0.9	-1.0	-1.0	-1.5	-1.5	gHBfix$_{opt}$	66%	27%	0.33	3.64	1.48	21%
0.3	0.8	-1.0	-1.0	-1.0	0.1	0.8	0.9	-1.0	0.0 !	-1.5	-1.5	gHBfix-constr (2'OH-2'OH constraint)	65%	24%	0.34	3.98	1.47	20%
0.5	0.7	-1.0	-1.0	-1.0	0.2	0.9	0.9	-1.0	0.0 !	-1.5	-1.5	gHBfix-constr$_{opt}$ (2'OH-2'OH constraint)	68%	25%	0.38	4.17	1.5	20%
0.3	0.8	-1.0	0.0 !	-1.0	0.1	0.8	0.9	-1.0	-1.0	-1.5	-1.5	gHBfix-constr (NH-2'OH constraint)	62%	18%	0.67	3.57	1.46	12%
0.5	0.7	-1.0	0.0 !	-1.0	0.1	0.8	0.9	-1.0	-1.0	-1.5	-1.5	gHBfix-constr$_{opt}$ (NH-2'OH constraint)	65%	19%	0.71	3.49	1.49	13%
0.3	0.8	-1.0	0.0 !	-1.0	0.1	0.8	0.9	-1.0	0.0 !	-1.5	-1.5	gHBfix-constr (*-2'OH constraint)	62%	14%	0.68	3.89	1.45	11%
0.7	0.7	-1.0	0.0 !	-1.0	0.1	0.8	0.9	-1.0	0.0 !	-1.5	-1.5	gHBfix-constr$_{opt}$ (*-2'OH constraint)	67%	15%	0.78	3.95	1.51	11%
0.3	0.5 !	-1.0	-1.0	-1.0	0.1	0.8	0.9	-1.0	-1.0	-1.5	-1.5	gHBfix-constr (W-C constraint)	58%	13%	0.27	3.41	1.64	5%
0.9	0.5 !	-1.0	-1.0	-1.0	-0.1	1.0	0.9	-1.0	-1.0	-1.5	-1.5	gHBfix-constr$_{opt}$ (W-C constraint)	66%	21%	0.43	3.83	1.65	13%

Figure S6: Performance of different sets of parameters where individual interactions have been manually constrained. We here report the performance predicted for both the training (first three columns, GACC, gcGAGAgc, gcUUCGgc) and validation (last three columns, CAAU, UUUU, gcUUCGgc) simulations, obtained with reweighting. The first two rows report the results corresponding to the previously published (gHBfix19) and optimized (gHBfix$_{opt}$) parameter sets. The following rows report the results corresponding to removing a single or a couple of correction terms (highlighted with an esclamation mark below the contact), either leaving the remaining terms unchanged (gHBfix-constr) or reoptimizing them (gHBfix-constr$_{opt}$). W-C constraints correspond to setting the NH-O bonds to a maximum of 0.5 kcal/mol, so as to limit the impact on the difference between the pairing energies associated to AU vs GC pairs. With exception of L1 stalk rRNA no training or validation system includes an AU W-C base pair and therefore we cannot rule out that the fitted parameters inaccurately affect the relative stability of AU or GC pairs. However, this can be compensated via readjusting the parameters of NH-O and NH-N interactions. In all the tested combinations, the effect of reoptimizing the parameters is mild, in agreement with what we observed in Fig. S1. A notable exception is the case where NH-O are set to 0.5 kcal/mol, where the reoptimization increases the attractive NH-N bonds to 0.9 kcal/mol to stabilize the Watson-Crick bonds in the duplex and recover the stability of the two tetraloops.

These tests are far from exhaustive. They however show that the reported test set and analysis tools can be easily used to assess the impact of individual changes to the force field, so as to predict the performance on the training set before running new simulations.

10 Explanation of provided scripts

The following subsections are labeled in the same way as the available scripts at `https://github.com/bussilab/ghbfix-training`. Even though all minimizations are performed on a rough landscape of the cost function, executing these scripts will result in the findings published and identical values for ideal hyperparameters and as a consequence gHBfix parameters. For the central identification of the regularization strength in the CV on observables the results found in this study are provided explicitly in order to follow the arguments made.

10.1 Supplementaries_CVTrajectory

This script requires that the script Supplementaries_CVTrajectory_PrepareBlocks has been executed previously, because it uses a data set split in predefined blocks in order to employ less memory at runtime. If the system on which these computations are performed possesses enough memory all 5 hyperparameter scans for each regularization penalty can be executed in parallel. In case one can not effort this amount of memory the processes can be run consecutively. This script is also used to partly produce the results of Fig. 4 in the main text.

10.2 Supplementaries_CVSystems

This script is used to produce the results of Fig. 5 in the main text. In order to execute this script efficiently the function main(), main1() and main2() can be executed once, because the error function is evaluated or minimized for all regularization penalties by construction. The functions main3() and main4() have to executed for each regularization penalty separately. Also here depending on the available memory of the system on which these computations are performed the processes can be run in parallel or consecutively.

10.3 Supplementaries_CVObservables

The cross-validation on observables is a central analysis for this study, because it results in the largest cross-validation error among the different protocols investigated for splitting the data. Executing the script should result in identical values for the ideal hyperparameter. However the scans published in this study are explicitly provided to follow the subsequent analysis. This script is additionally used to partly produce the results of Fig. 4 in the main text.

10.4 Supplementaries_GetParameters

Running this script will load and fit the full training dataset with the optimal regularization penalty and hyperparameter identified during cross-validation on observables. This script is additionally used to produce the results of Fig. 2 and Fig. 6 in the main text.

10.5 Supplementaries_FitYourself

This script can be used to obtain gHBfix parameters in which corrections for selected contacts (e.g. the 2′OH-2′OH or the NH-2′OH) are omitted. This script can also be used to find new parameters by specifying boundaries instead. In this case one initializes an empty array called 'which_interaction_index_to_exclude' and customize the array 'bounds' to satisfy any custom constraint. For example the Watson-Crick pairing interaction can be constrained. All these alternative force fields and their effects on systems can be seen in Fig. S6.

10.6 Supplementaries_ComputeErrorsAndPopulations

This script is used to produce the results of Table 1 of the main text.

10.7 Supplementaries_IndivParam0

This script is used to produce the results of Fig. S1.

10.8 Supplementaries_FavoringInteractionsFigures

This script is used to produce the results of Fig. S2.

10.9 Supplementaries_ErrorEstimate

This script is used to compute the error estimates of the native population in Table 1 of the results in the main text. It can be used to estimate the error with bootstrap on any observable of the training and validation set.

11 Parameter sets of cross-validation on systems

In Tables S3-S6 all parameters associated to the analysis of Fig. 6 in the main text are collected.

Table S3: Parameters for gHBfix proton donor and acceptor corrections associated the cross-validation on systems in which GACC is the validation set with and without regularization are indicated. When using regularization, parameters are extracted at the hyperparameter indicated with a star in the cross-validation plots Fig. 6 and expressed as $k_B T \cdot \lambda$ in kcal/mol.

	**No regularization**		**Regularization (Relative Kish Size)**	
Acceptor/Donor	NH	2'OH	NH	2'OH
N	0.5	1.4	0.3	1.0
O	1.1	1.1	0.8	0.8
O4'	-0.4	-1.0	-1.0	-1.0
2'OH	-1.7	-1.0	-1.0	-1.0
bO	-0.6	-2.1	-1.0	-1.3
nbO	0.2	-1.0	0.2	-1.3

Table S4: Parameters for gHBfix proton donor and acceptor corrections associated the cross-validation on systems in which gcGAGAgc is the validation set with and without regularization are indicated. When using regularization, parameters are extracted at the hyperparameter indicated with a star in the cross-validation plots Fig. 6 and expressed as $k_B T \cdot \lambda$ in kcal/mol.

	**No regularization**		**Regularization (Relative Kish Size)**	
Acceptor/Donor	NH	2'OH	NH	2'OH
N	-0.3	0.8	-0.1	0.9
O	1.1	0.9	0.8	0.9
O4'	-34.2	-114.8	-1.0	-1.0
2'OH	-86.7	-33.5	-1.0	-1.0
bO	-44.6	-48.4	-1.0	-1.3
nbO	0.0	-0.1	0.1	-1.3

Table S5: Parameters for gHBfix proton donor and acceptor corrections associated the cross-validation on systems in which gcUUCGgc is the validation set with and without regularization are indicated. When using regularization, parameters are extracted at the hyperparameter indicated with a star in the cross-validation plots Fig. 6 and expressed as $k_B T \cdot \lambda$ in kcal/mol.

	**No regularization**		**Regularization (Relative Kish Size)**	
Acceptor/Donor	NH	2'OH	NH	2'OH
N	1.3	-0.1	-0.4	0.4
O	0.1	-14.7	0.8	-1.0
O4'	-11.0	-1.5	-1.0	-1.0
2'OH	-30.0	-9.3	-1.0	0.2
bO	-6.6	-6.3	-1	-1.3
nbO	0.1	0.0	0.2	-1.3

Table S6: Parameters for gHBfix proton donor and acceptor corrections associated the fitting performed on all systems (GACC,gcGAGAgc and gcUUCGgc) simultaneously with and without regularization are indicated. When using regularization, parameters are extracted at the hyperparameter indicated with a star in the cross-validation plots Fig. 6 and expressed as $k_B T \cdot \lambda$ in kcal/mol.

	**No regularization**		**Regularization (Relative Kish Size)**	
Acceptor/Donor	NH	2'OH	NH	2'OH
N	0.1	0.8	0.3	0.8
O	1.1	0.9	0.8	0.9
O4'	-46.3	-114.0	-1.0	-1.0
2'OH	-89.9	-32.3	-1.0	-1.0
bO	-44.6	-53.3	-1.0	-1.5
nbO	0.0	-0.3	0.1	-1.5

References

(1) Kührová, P.; Mlỳnskỳ, V.; Zgarbová, M.; Krepl, M.; Bussi, G.; Best, R. B.; Otyepka, M.; Šponer, J.; Banáš, P. Improving the performance of the AMBER RNA force field by tuning the hydrogen-bonding interactions. *J. Chem. Theory Comput.* **2019**, *15*, 3288–3305.

(2) Bottaro, S.; Di Palma, F.; Bussi, G. The role of nucleobase interactions in RNA structure and dynamics. *Nucleic Acids Res.* **2014**, *42*, 13306.

(3) Bottaro, S.; Bussi, G.; Pinamonti, G.; Reißer, S.; Boomsma, W.; Lindorff-Larsen, K. Barnaba: software for analysis of nucleic acid structures and trajectories. *RNA* **2019**, *25*, 219–231.

(4) Condon, D. E.; Kennedy, S. D.; Mort, B. C.; Kierzek, R.; Yildirim, I.; Turner, D. H. Stacking in RNA: NMR of Four Tetramers Benchmark Molecular Dynamics. *J. Chem. Theory Comput.* **2015**, *11*, 2729–2742.

(5) Zhao, J.; Kennedy, S. D.; Berger, K. D.; Turner, D. H. Nuclear Magnetic Resonance of Single-Stranded RNAs and DNAs of CAAU and UCAAUC as Benchmarks for Molecular Dynamics Simulations. *J. Chem. Theory Comput.* **2020**, *16*, 1968–1984.

(6) Bottaro, S.; Bussi, G.; Kennedy, S. D.; Turner, D. H.; Lindorff-Larsen, K. Conformational ensembles of RNA oligonucleotides from integrating NMR and molecular simulations. *Science Adv.* **2018**, *4*, eaar8521.

(7) Condon, D. E.; Kennedy, S. D.; Mort, B. C.; Kierzek, R.; Yildirim, I.; Turner, D. H. Stacking in RNA: NMR of Four Tetramers Benchmark Molecular Dynamics. *J. Chem. Theory Comput.* **2015**, *11*, 2729.

(8) Lankhorst, P.; Haasnoot, C. A. G.; Erkelens, C.; Westerink, H.; Marel, G.; Boom, J.; Altona, C. Carbon-13 NMR in conformational analysis of nucleic acid fragments. 4. The torsion angle distribution about the C3'-O3' bond in DNA constituents. *Nucleic Acids Res.* **1985**, *13*, 927–42.

(9) Davies, D. B. Conformations of nucleosides and nucleotides. *Prog. Nucl. Magn. Reson. Spectrosc.* **1978**, *12*, 135–225.

(10) Bonomi, M.; Bussi, G.; Camilloni, C.; Tribello, G. A.; Banáš, P.; Barducci, A.; Bernetti, M.; Bolhuis, P. G.; Bottaro, S.; Branduardi, D.; Capelli, R.; Carloni, P.; Ceriotti, M.; Cesari, A.; Chen, H.;

Chen, W.; Colizzi, F.; De, S.; De La Pierre, M.; Donadio, D.; Drobot, V.; Ensing, B.; Ferguson, A. L.; Filizola, M.; Fraser, J. S.; Fu, H.; Gasparotto, P.; Gervasio, F. L.; Giberti, F.; Gil-Ley, A.; Giorgino, T.; Heller, G. T.; Hocky, G. M.; Iannuzzi, M.; Invernizzi, M.; Jelfs, K. E.; Jussupow, A.; Kirilin, E.; Laio, A.; Limongelli, V.; Lindorff-Larsen, K.; Löhr, T.; Marinelli, F.; Martin-Samos, L.; Masetti, M.; Meyer, R.; Michaelides, A.; Molteni, C.; Morishita, T.; Nava, M.; Paissoni, C.; Papaleo, E.; Parrinello, M.; Pfaendtner, J.; Piaggi, P.; Piccini, G.; Pietropaolo, A.; Pietrucci, F.; Pipolo, S.; Provasi, D.; Quigley, D.; Raiteri, P.; Raniolo, S.; Rydzewski, J.; Salvalaglio, M.; Sosso, G. C.; Spiwok, V.; Šponer, J.; Swenson, D. W. H.; Tiwary, P.; Valsson, O.; Vendruscolo, M.; Voth, G. A.; White, A. Promoting transparency and reproducibility in enhanced molecular simulations. *Nat. Methods* **2019**, *16*, 670–673.

(11) Mlynsky, V.; Janecek, M.; Kuhrova, P.; Frohlking, T.; Otyepka, M.; Bussi, G.; Banas, P.; Sponer, J. Toward Convergence in Folding Simulations of RNA Tetraloops: Comparison of Enhanced Sampling Techniques and Effects of Force Field Modifications. *J. Chem. Theory Comput.* **2022**, *18*, 2642–2656.

(12) Nozinovic, S.; Fürtig, B.; Jonker, H. R.; Richter, C.; Schwalbe, H. High-resolution NMR structure of an RNA model system: the 14-mer cUUCGg tetraloop hairpin RNA. *Nucleic Acids Res.* **2010**, *38*, 683.

(13) Kührová, P.; Mlỳnskỳ, V.; Zgarbová, M.; Krepl, M.; Bussi, G.; Best, R. B.; Otyepka, M.; Šponer, J.; Banáš, P. Correction to "Improving the performance of the AMBER RNA force field by tuning the hydrogen-bonding interactions". *J. Chem. Theory Comput.* **2020**, *16*, 818–819.

(14) Klein, D.; Schmeing, T.; Moore, P.; Steitz, T. The kink-turn: a new RNA secondary structure motif. *EMBO J.* **2001**, *20*, 4214–4221.

(15) Schroeder, K.; McPhee, S.; Ouellet, J.; Lilley, D. A structural database for k-turn motifs in RNA. *RNA* **2010**, *16*, 1463–8.

(16) Huang, L.; Lilley, D. The Kink Turn, a Key Architectural Element in RNA Structure. *J. Mol. Biol.* **2015**, *428*.

(17) Liu, J.; Lilley, D. The role of specific 2′-hydroxyl groups in the stabilization of the folded conformation of kink-turn RNA. *RNA* **2007**, *13*, 200–10.

(18) Rázga, F.; Koča, J.; Šponer, J.; Leontis, N. B. Hinge-Like Motions in RNA Kink-Turns: The Role of the Second A-Minor Motif and Nominally Unpaired Bases. *Biophys. J.* **2005**, *88*, 3466–3485.

(19) Rázga, F.; Zacharias, M.; Réblová, K.; Koca, J.; Sponer, J. RNA Kink-Turns as Molecular Elbows: Hydration, Cation Binding, and Large-Scale Dynamics. *Structure* **2006**, *14*, 825–35.

(20) Curuksu, J.; Sponer, J.; Zacharias, M. Elbow Flexibility of the kt38 RNA Kink-Turn Motif Investigated by Free-Energy Molecular Dynamics Simulations. *Biophys. J.* **2009**, *97*, 2004–13.

(21) Goody, T.; Melcher, S.; Norman, D.; Lilley, D. The kink-turn motif in RNA is dimorphic, and metal ion-dependent. *RNA* **2004**, *10*, 254–64.

(22) Huang, L.; Lilley, D. M. J. The kink-turn in the structural biology of RNA. *Q. Rev. Biophys.* **2018**, *51*, e5.

(23) Krepl, M.; Réblová, K.; Koca, J.; Sponer, J. Bioinformatics and Molecular Dynamics Simulation Study of L1 Stalk Non-Canonical rRNA Elements: Kink-Turns, Loops, and Tetraloops. *J. Phys. Chem. B* **2013**, *117*.

(24) Réblová, K.; Šponer, J. E.; Špačková, N.; Beššeová, I.; Šponer, J. A-Minor Tertiary Interactions in RNA Kink-Turns. Molecular Dynamics and Quantum Chemical Analysis. *J. Phys. Chem. B* **2011**, *115*, 13897–13910.

(25) Daldrop, P.; Lilley, D. M. The plasticity of a structural motif in RNA: Structural polymorphism of a kink turn as a function of its environment. *RNA* **2013**, *19*, 357–364.

(26) Gao, Y. G.; Selmer, M.; Dunham, C. M.; Weixlbaumer, A.; Kelley, A. C.; Ramakrishnan, V. The structure of the ribosome with elongation factor G trapped in the posttranslocational state. *Science* **2009**, *326*, 694–699.

(27) Cornish, P. V.; Ermolenko, D. N.; Staple, D. W.; Hoang, L.; Hickerson, R. P.; Noller, H. F.; Ha, T. Following movement of the L1 stalk between three functional states in single ribosomes. *Proc. Natl. Acad. Sci. U.S.A.* **2009**, *106*, 2571–2576.

(28) Tishchenko, S.; Gabdulkhakov, A.; Nevskaya, N.; Sarskikh, A.; Kostareva, O.; Nikonova, E.; Sycheva, A.; Moshkovskii, S.; Garber, M.; Nikonov, S. High-resolution crystal structure of the isolated ribosomal L1 stalk. *Acta Crystallogr. D* **2012**, *68*, 1051–1057.

(29) Trabuco, L. G.; Schreiner, E.; Eargle, J.; Cornish, P.; Ha, T.; Luthey-Schulten, Z.; Schulten, K. The role of L1 stalk-t RNA interaction in the ribosome elongation cycle. *J. Mol. Biol.* **2010**, *402*, 741–760.

(30) Sakuraba, S.; Asai, K.; Kameda, T. Predicting RNA Duplex Dimerization Free-Energy Changes upon Mutations Using Molecular Dynamics Simulations. *J. Phys. Chem. Lett.* **2015**, *6*, 4348–4351.

(31) Sakuraba, S.; Iwakiri, J.; Hamada, M.; Kameda, T.; Tsuji, G.; Kimura, Y.; Abe, H.; Asai, K. Free-Energy Calculation of Ribonucleic Inosines and Its Application to Nearest-Neighbor Parameters. *J. Chem. Theory Comput.* **2020**, *16*, 5923–5935.
